# Spatial Reorganization of Chromatin Architecture Shapes the Expression Phenotype of Therapy‐Induced Senescent Cells

**DOI:** 10.1111/acel.70366

**Published:** 2026-01-06

**Authors:** Ge Zhang, Wei Zhang, Changxu Wang, Zhirui Jiang, Qixia Xu, Haipeng Li, James L. Kirkland, Gang Wei, Yu Sun

**Affiliations:** ^1^ Shanghai Institute of Nutrition and Health, Chinese Academy of Sciences Shanghai China; ^2^ Center for Single‐Cell Omics, School of Public Health, Shanghai Jiao Tong University School of Medicine Shanghai China; ^3^ Center for Advanced Gerotherapeutics, Cedars‐Sinai Medical Center, Pacific Design Center West Hollywood California USA; ^4^ Division of Endocrinology, Diabetes and Metabolism Cedars‐Sinai Medical Center Los Angeles California USA; ^5^ Key Laboratory of Molecular Virology and Immunology Shanghai Institute of Immunity and Infection, Shanghai Institute of Materia Medica, Chinese Academy of Sciences Shanghai China; ^6^ School of Pharmacy, Institute of Aging Medicine Binzhou Medical University Yantai China

## Abstract

Cellular senescence is a fundamental biological process contributing to aging, often accompanied by extensive chromatin remodeling. Dynamic alterations of three‐dimensional (3D) genomic spatial structure, driven by chromatin reorganization, play a critical role in cell fate determination, but their relevance in therapy‐induced senescence (TIS) remains underexplored. Here, we perform an integrative multi‐omics analysis of Hi‐C, ATAC‐seq, CUT&RUN, and RNA‐seq in primary human fibroblasts undergoing TIS induced by ionizing radiation (RAD) or bleomycin (BLEO). We show that TIS leads to global chromatin decompaction, weakened compartmentalization, and destabilization of topologically associated domains (TADs), alongside widespread loss and rewiring of chromatin loops. Notably, RAD and BLEO elicit distinct changes in distance‐dependent compartment strength and enhancer–promoter (E‐P) loop patterns, reflecting divergent 3D regulatory programs. Importantly, TIS reshapes the chromatin environment around senescence‐associated secretory phenotype (SASP) genes, while their adjacent regions exhibit reduced chromatin interactions, allowing transcriptional activation. Our study reveals that 3D genome remodeling in TIS is highly plastic and context‐dependent and discloses spatial regulation of gene expression during therapy‐induced cellular senescence.

## Graphical abstract

In the course of TIS, cells undergo a profound epigenomic reorganization that underlies the development of a senescence‐associated phenotype and formation of an inflammatory microenvironment.

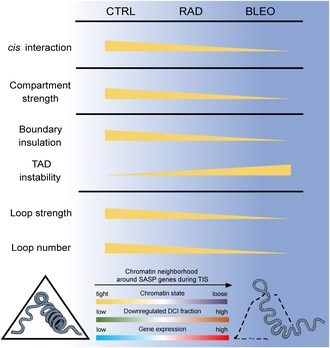

## 1 Introduction

Given the impact of cellular senescence on chronological aging, an unprecedented pressure on global healthcare, an insightful understanding of senescence per se is important. Cellular senescence was originally identified in normal human fibroblasts as a result of consecutive growth and exhaustion of proliferative potential, named as replicative senescence (RS) (Hayflick and Moorhead 1961). RS is primarily driven by telomere shortening and/or telomere dysfunction, which triggers DNA damage responses and compromises genomic integrity in subtelomeric regions (Harley et al. 1990). Alternatively, primary cells exhibit a similar growth arrest in response to oncogene activation, a case usually referred to as oncogene‐induced senescence (OIS) (Serrano et al. 1997). Oncogenic signals, such as activated RAS, can paradoxically induce a stable senescence program despite promoting proliferation and survival in other contexts. Ectopic expression of RAS in human and mouse fibroblasts results in a flattened morphology and an essentially irreversible G1 arrest, accompanied by accumulation of canonical senescence regulators, including p53 and p16 (Serrano et al. 1997). Senescence‐associated phenomena were then further extended to processes causing cell‐cycle arrest in response to diverse forms of insults such as oxidative stress, chemotherapy, and ionizing radiation (Campisi and di Fagagna 2007; Di Micco et al. 2011). Senescent cell‐exerted in vivo effects are primarily mediated by the development of the senescence‐associated secretory phenotype (SASP), the composition of which varies depending on specific cell types, senescence inducers, and microenvironment settings (McHugh et al. 2025). The SASP components, mostly identified in human diploid fibroblast (HDF) models, include pro‐inflammatory cytokines, chemokines, metalloproteinases, and extracellular matrix remodeling factors (Du et al. 2025).

The structure of chromatin is subject to precise regulation by epigenetic modifications such as DNA methylation and histone posttranslational modification (PTM), changes that functionally govern gene expression via chromatin organization and genome accessibility. Indeed, the epigenetic landscape can experience perpetual fluctuations throughout the lifespan and is profoundly altered with aging (Gadecka and Bielak‐Zmijewska 2019). Perturbations of histone methylation‐associated marks, such as loss of H3K9me3 and H3K27me3, can occur during normal human aging and premature aging diseases, such as Hutchinson–Gilford progeria syndrome or Werner syndrome, implying global decline of heterochromatin as a potentially common feature of aging (Gruenbaum and Foisner 2015). Although RS and OIS cells share a few common features, including DNA damage, morphological changes, activation of p53/p21 and p16/Rb pathways, as well as induction of the SASP, they differ remarkably in nuclear architecture. OIS nuclei frequently display heterochromatin bodies, called senescence‐associated heterochromatin foci (SAHF), enriched in H3K9me3 and other core heterochromatin marks across different human cell types and pathologic conditions, while RS nuclei are enlarged and display a variety of features, such as compaction of individual chromosome arms and distension of peri‐centromeric regions (Chandra et al. 2012; Cruickshanks et al. 2013; De Cecco et al. 2013). These dramatic chromatin alterations typically occur in RS and OIS cells, with a capacity to affect nuclear architecture. Both radiotherapy and chemotherapy can also elicit cellular senescence, a phenomenon termed therapy‐induced senescence (TIS). Common inducers include doxorubicin, bleomycin, paclitaxel, platinum‐based agents, alkylating drugs, and ionizing radiation. Notably, lower‐dose regimens more often promote senescence, whereas higher‐dose exposure can shift the cellular outcome toward apoptosis (Wang et al. 2020; Chibaya et al. 2022; Wang et al. 2022). Mechanistically, TIS is largely attributed to the accumulation of therapy‐induced genomic lesions; certain chemotherapeutics additionally increase reactive oxygen species and/or suppress telomerase activity, thereby reinforcing senescence programs and potentially accelerating cellular aging.

Several well‐developed high‐throughput chromosome conformation capture approaches provide a platform to realize a high‐resolution interrogation of chromosome structure and to explore the relationship between 3D chromosomal architecture and gene expression (Kempfer and Pombo 2020). Hi‐C experiments revealed a fundamental and large‐scale organization of the genome into two types of compartments, namely A and B (Zhao et al. 2024). Being approximately 5 Mb in size and cell type‐specific, the A compartments basically correlate with early replicating euchromatic regions, while the B compartments are associated with heterochromatin (Zhao et al. 2024). The genome is organized into topologically associated domains (TADs) identifiable by frequent interactions between loci within or across domains, a feature that can be found at higher resolutions (< 2 Mb) (Baudic et al. 2024). TADs are located in either A or B compartments, while repositioning between A and B compartments causes coordinated expression changes of genes within switching compartments and can play important roles during cell differentiation and cell fate determination (Ma et al. 2024). However, TADs are highly conserved across cell lineages and even across species, and may constitute a basic organizational unit of the genome (Phillips‐Cremins et al. 2013). The 3D chromatin organization in TADs and chromatin loops, mediating interactions of regulatory elements such as enhancers and promoters, is critical for tissue‐specific gene regulation (Baudic et al. 2024). In addition, super‐resolution microscopy has revealed an interchromatin compartment (IC). Active genes and soluble chromatin regulatory factors are frequently found at the surfaces of chromatin domains (CDs) and within the IC.

Although advanced technologies have increased our understanding of 3D genomic organization (Liu et al. 2022; Olan et al. 2020; Criscione et al. 2016; Zhang et al. 2023; Rao et al. 2014), insights into higher‐order chromatin modifications in distinct forms of senescence remain yet limited. A former study applying low resolution Hi‐C to examine early stages of OIS found no changes in TAD borders, while another report focusing on RS showed alterations in TAD borders and switching of some TADs from active to inactive compartments (Criscione et al. 2016; Chandra et al. 2015). Experimental data indicated a shift in the ratio between short‐ and long‐range chromatin contacts, but they do not consistently support the direction of such a shift. Hi‐C and Micro‐C profiling have revealed extensive 3D genome reorganization during RS and OIS, including alterations in compartment, domain‐level organization, and chromatin looping (Zirkel et al. 2018; Sofiadis et al. 2021; Palikyras et al. 2024). Although 3D genome changes have also been examined in TIS triggered by bleomycin or ionizing radiation, these two genotoxic TIS models have not been interrogated side‐by‐side within a single study, and the extent to which TIS‐associated 3D chromatin remodeling is coupled to transcriptional programs remains insufficiently defined. Recent advances in Hi‐C technologies have unmasked additional structural units, such as chromatin loops and enhancer–promoter (EP) contacts (Schoenfelder and Fraser 2019; Javierre et al. 2016). However, how 3D genome organization influences transcriptional regulators, how it spatiotemporally dictates global expression pattern, and whether or not distinct mechanisms are engaged in senescent cells, particularly in the case of TIS, remains largely unknown. In this study, we addressed key issues by employing human HDFs to further investigate TIS, with a major focus on establishing fundamental features of the 3D genome organization of TIS cells. We integrated Hi‐C, ATAC‐seq, CUT&RUN, and bulk RNA sequencing (RNA‐seq) with TIS cells, and dissected general relationships between 3D genomic organization and transcriptional regulation of SASP genes during senescence.

## 2 Results

### 2.1 HDFs Undergoing TIS Exhibit Typical Senescence‐Associated Phenotypic Changes

**FIGURE 1:**
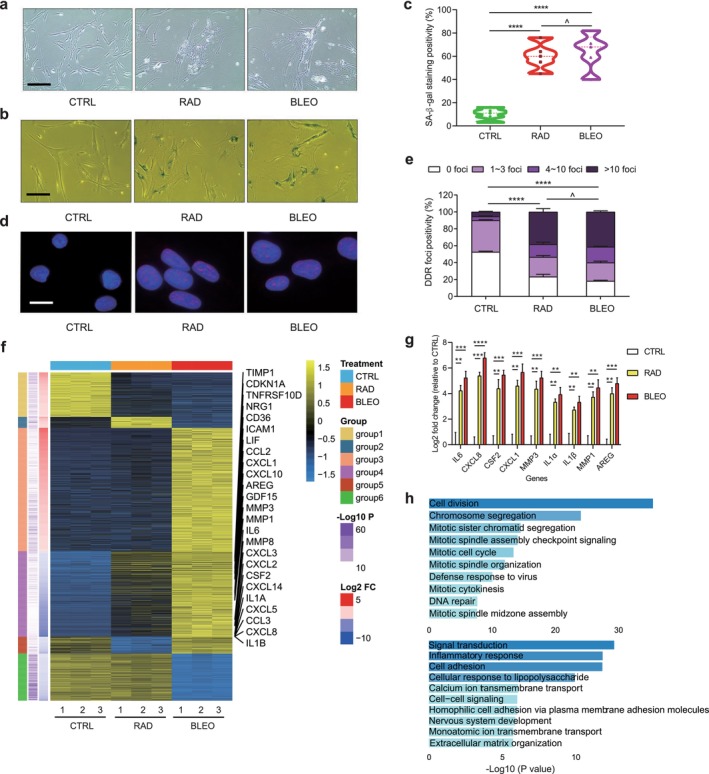
Therapy‐induced senescence and global expression profile of human diploid fibroblasts. (a) Representative phase contrast images of human primary prostate fibroblast cells of PSC27 line after treatment by either ionizing radiation (RAD, 10 Gy) or bleomycin (BLEO, 50 μg/mL). Scale bar, 20 μm. (b) Senescence assessment with SA‐β‐Gal staining of fibroblasts as described in a. Representative images are shown. Scale bars, 20 μm. (c) Comparative statistics of senescence positivity upon SA‐β‐Gal staining as described in b. (d) Examination of DNA damage response (DDR) by immunofluorescence (IF) staining against γH2AX (red) and counterstaining with DAPI (blue). Representative images are shown. Scale bars, 5 μm. (e) Comparative statistics of DDR profiling upon IF staining as described in d. (f) Heatmap of treatment‐specific differential expression genes (DEGs) ordered by log_2_(fold change) per group. The selected SASP factors are shown on the right. DEGs are filtered by adjusted *p*‐value < 0.01 and |log_2_(fold change)| > 1. (g) Quantitative measurement of the expression of typical SASP factors. (h) Bar plot showing the top 10 GO terms of DEGs from group 1 (top) and group 4 (bottom), respectively. TIS, therapy‐induced senescence. ^*p* > 0.05; **p < 0.01; ***p < 0.001; ****p < 0.0001.

A primary normal HDF line PSC27, was chosen for senescence‐associated experiments in this study. PSC27 expresses and secretes a large array of SASP factors upon senescence induced by environmental or inherent stresses (Sun et al. 2012; Zhang et al. 2018). After exposure to ionizing radiation (RAD) or bleomycin (BLEO), the latter is a chemotherapeutic agent widely used for cancer patients in clinical settings, PSC27 displayed remarkable changes in cell morphology in culture (Figure 1a). After treatment by either RAD or BLEO, cells displayed elevated DNA damage response (DDR) and entered a typical senescence status, as evidenced by increased senescence‐associated beta‐galactosidase (SA‐β‐Gal) staining positivity (Figure 1b–e) and decreased BrdU incorporation (Figure S1a). Immunoblot analyses verified upregulated expression of several key protein molecules, including classic SASP factors MMP3, IL6, and CXCL8 (Figure S1b). Data from bulk RNA sequencing (RNA‐seq, triplicate per treatment modality) indicated a substantial alteration of the global expression pattern of PSC27 cells upon senescence under in vitro treatment conditions. We defined six groups of differentially expressed genes (DEGs) based on specific up‐ or downregulation patterns observed in CTRL, RAD, and BLEO. These groups comprised 458, 114, 1271, 882, 173, and 486 DEGs, respectively. Among these genes, the 1st group (*n* = 458) represented genes commonly downregulated by both RAD and BLEO treatments, whereas the 4th group (*n* = 882) consisted of genes commonly upregulated by both senescence‐inducing modalities (Figure 1f). We then compared each of the six DEG sets with the SenMayo senescence gene set (Saul et al. 2022). Among them, the 4th group showed the strongest association with the SenMayo gene set, indicating that this group most robustly captures a canonical senescence‐associated transcriptional program (Figure S1c). Furthermore, we observed that numerous SASP factors exhibited elevated expression levels in senescent cells. In addition, the expression of neuregulin 1 (NRG1), a membrane glycoprotein that mediates intercellular signaling and regulates the growth and development of multiple organs, was also considerably enhanced in senescent cells (Figure 1f). Beyond transcriptome‐wide RNA‐seq, we performed quantitative RT‐PCR assays and found significantly elevated expression of several hallmark SASP factors (Figure 1g).

To investigate the functional alterations associated with TIS, we performed Gene Ontology (GO) enrichment analysis of DEGs in the 1st and 4th groups (Figure 1h). The senescent downregulated genes were predominantly enriched in biological processes related to cell cycle regulation, including cell division, chromosome segregation, mitotic spindle assembly, and checkpoint signaling. These findings indicate a profound inhibition of proliferative capacity and cell cycle progression, highlighting cell cycle arrest as a hallmark of TIS. In contrast, senescent upregulated genes were largely associated with biological processes involved in signal transduction, inflammatory response, cell adhesion, and cellular immune responses. This shift suggests that TIS occurrence is accompanied by the activation of inflammatory signaling, enhanced cell–microenvironment interactions, and remodeling of the extracellular milieu, which may collectively contribute to the establishment of a pro‐inflammatory senescent microenvironment.

### 2.2 Spatial Genome Remodeling in TIS is Mediated by Distinct Interactions Between Chromatin Compartments

To establish TIS‐associated spatial organization of 3D genome architecture, we dissected stress‐exposed PSC27 cells with Hi‐C, using two independent biological replicates per condition. Each Hi‐C assay was performed by sequencing of 580 to 777 million paired‐end reads, generating 249 to 392 million unique valid pairs (Table S1). Sequencing reads were first aligned to the human genome (GRCh37/hg19) by iterative mapping, then filtered, binned, and corrected for biases using iterative correction and eigenvector decomposition (ICE). To check the resolution availability, we quantified the effective Hi‐C resolution as the smallest bin size at which ≥ 80% of genomic bins have at least 500 or 1000 unique contacts. The sequencing depth of this study provides sufficient coverage to robustly resolve higher‐order chromatin features, enabling reliable detection of A/B compartments, TADs, and chromatin loops (Figure S1d). As doses used for cell stress treatments were based on priori of dose responses, the damage responses we observed are representative of typical cellular responses under in vitro conditions, with cell passage numbers controlled relatively early as compared with those of RS cells. The values in the Hi‐C matrix represent the frequency of interactions between bins. After data quality and reproducibility checks, we combined two biological replicates for each condition (Figure S1e–g). To compare across different conditions, we then down‐sampled Hi‐C datasets to 340 million at the fragment level to ensure the contribution of an equal number of reads to each Hi‐C dataset matrix.

**FIGURE 2:**
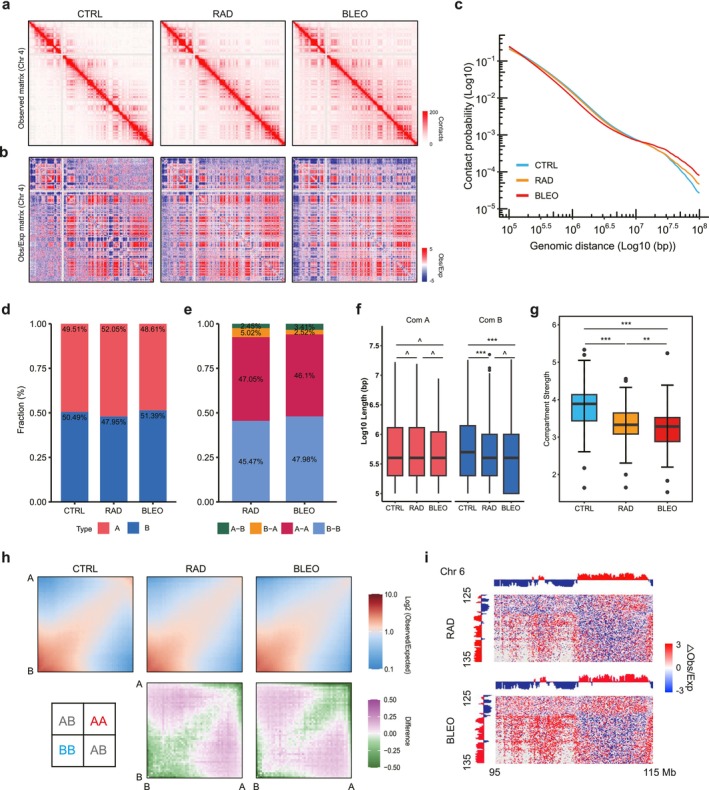
TIS leads to whole‐genome interaction changes. (a) Observed heatmap of chromosome 11 per sample. (b) Observed/expected heatmap of chromosome 11 per sample. (c) The chromatin contact probabilities (*P*(s)) relative to genomic distance for CTRL, RAD, and BLEO, respectively. (d) The proportion of A/B compartments of each sample. (e) Fraction of compartment‐switched regions including A–A, B–B, A–B, B–A. (f) Boxplot of A/B compartment length. The *p* values were calculated by unpaired Wilcoxon tests. ^*p* > 0.05; *p < 0.05; **p < 0.01; ***p < 0.001. (g) Boxplot of compartment strength. The *p* values were calculated by paired *t*‐tests. ^*p* > 0.05; *p < 0.05; **p < 0.01; ***p < 0.001. (h) Saddle plots and differential saddles are displayed. Bins are assigned to fifty deciles based on PC1 values; average observed/expected distance‐normalized scores for each pair of deciles are simultaneously calculated. (i) Differential heatmaps showing the regions on chromosome 6 corresponding to the change of intra‐ and inter‐interactions in RAD/BLEO versus CTRL. The PC1 values for RAD and BLEO are also shown.

Induction of cellular senescence in HDF via ionizing radiation or bleomycin treatment resulted in discernible alterations to the 3D chromatin architecture. The *cis* interaction fractions in CTRL, RAD, and BLEO conditions were 90.34%, 83.32%, and 70.9%, respectively, indicating the association of TIS with extensive chromatin reorganization and a concomitant loss of local chromatin compaction (Figure S2a–g). The Hi‐C matrix displayed a classical checkerboard‐like pattern. The potential changes in higher‐order chromatin structures upon TIS appear to be shifts in chromatin compartment organization and a weakening or blurring of TAD boundaries (Figure 2a,b). To investigate the distribution of intra‐chromosomal contacts in detail and to exclude distance effects, we performed an examination of relative contact probabilities (RCP) with respect to genomic distance. The mean contact probabilities of proliferating and senescent cells were largely consistent with a power law, as previously observed (Criscione et al. 2016). The reduction of interaction enrichment was observed at short‐ and medium‐range distances, whereas an increase of interactions was evident at long‐range distances (> ~30 Mb), indicating a redistribution of chromatin interactions across different spatial scales during TIS (Figures 2c and S2h,i). The significant genome‐wide spatial reorganization accompanying the establishment of the senescence state can alter the epigenetic landscapes (Figure S3), consistent with previously characterized large‐scale chromatin remodeling events associated with senescence, which may in turn exert functional impacts on gene regulation (Liu et al. 2022).

### 2.3 Global Compartmentalization Tends to be Disordered in Cells Undergoing TIS

To further define the chromatin structure patterns, we next examined the compartment level of TIS. In contrast to the extensive compartmental reorganization observed during oncogene‐induced senescence (OIS) in IMR90 human fibroblasts as reported previously (Iwasaki et al. 2019), we found that the proportion of A and B compartments remained approximately 50% each across all 3 conditions in the case of TIS. Compared to non‐senescent cells, only ~7.5% and ~6% of genomic regions underwent compartment switching in RAD and BLEO, respectively. The length of A compartments remained largely unchanged, whereas B compartments appeared to be shorter in senescent cells. This subtle compartmental change pattern suggests that TIS does not cause large‐scale alterations in the A/B compartment switch (Figure 2d–f).

We next quantitatively assessed genome compartmentalization. There was a marked decrease in overall compartmentalization strength under both TIS conditions (Figure 2g and Table S2). Consistent with this, saddle plots derived from Hi‐C data at 100 kb resolution, particularly differential saddle plots, revealed significantly elevated interaction frequencies between normally segregated active A and repressive B compartments in RAD and BLEO samples. This signifies a profound disruption of chromatin higher‐order structure during TIS. Strikingly, despite this commonality, RAD and BLEO treatments imprinted opposing signatures on intra‐compartment interactions. RAD treatment resulted in a significant decrease in BB interaction frequency while slightly increasing AA interactions. Conversely, BLEO treatment promoted BB interactions alongside a stronger reduction in AA interactions, highlighting therapy‐specific alterations in genome topology during senescence (Figures 2h,i and S2j). While both RAD and BLEO induce senescence via the generation of DDR, their differing modes of damage induction result in variations in chromatin higher‐order organization.

**FIGURE 3:**
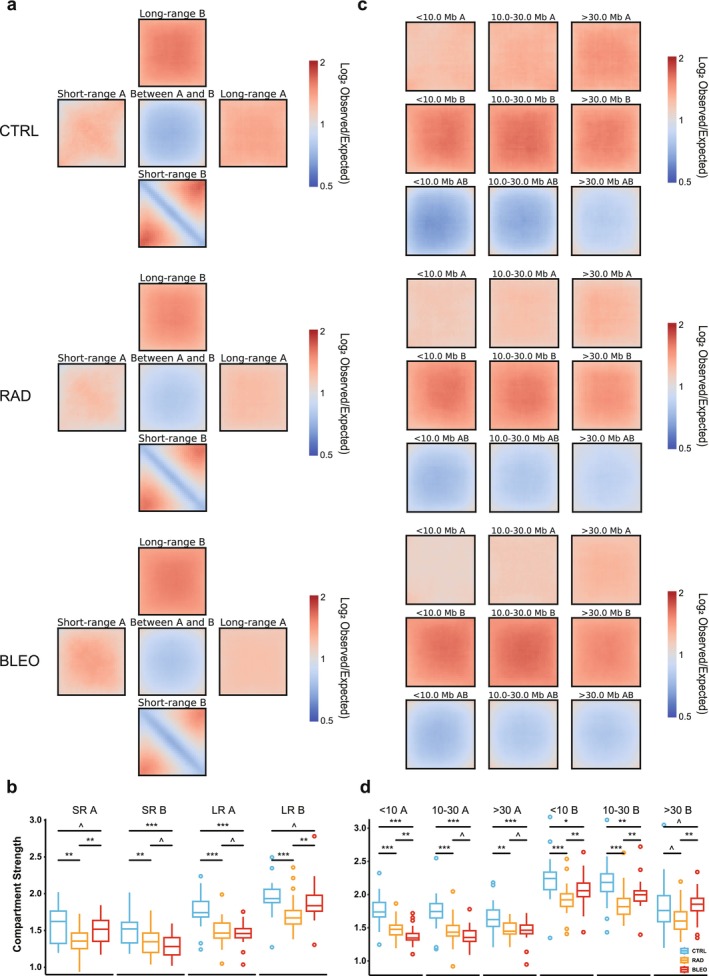
Distance‐dependent compartment strength alterations during TIS. (a) Heatmaps depicting the long‐ and short‐range distance compartmentalization. (b) Boxplots of quantification of compartment strength for different types of compartment interactions. SR, short range; LR, long range. (c) Compartmentalization heatmaps per sample at different genomic distance ranges. (d) Quantification of the compartment strength over different genomic distance ranges. In b and d, the *p* values between treatments were calculated by paired Wilcoxon tests. ^*p* > 0.05; *p < 0.05; **p < 0.01; ***p < 0.001.

Conventional saddle plot analysis involves ordering genomic regions based on their PC1 values, partitioning them into bins, and subsequently calculating the corresponding interaction frequencies between these bins. Notably, this standard approach does not explicitly account for the factor of linear genomic distance. Therefore, to interpret compartmentalization changes of all 3 groups in further details, we chose to use Pentad, a distance‐dependent tool, for quantification of chromatin interactions within and between compartments (Magnitov et al. 2022) (Figure S4a). We first analyzed average interaction frequencies and compartment strength within and between A/B compartments, differentiating between short‐range (regions on the Hi‐C heatmap diagonal) and long‐range (regions off the Hi‐C heatmap diagonal) interactions. Compared to CTRL, RAD led to a reduction in both short‐ and long‐range A/B compartment‐associated interactions, although the extent of reduction varied. In contrast, BLEO treatment exhibited a distinct pattern, characterized by a specific loss of short‐range (SR) interactions involving B compartments and long‐range (LR) interactions involving A compartments (Figures 3a and S4b). Across short‐ and long‐range interactions within both A and B compartments, RAD showed significantly weaker compartmentalization than CTRL, whereas BLEO exhibited significantly stronger compartmentalization in short‐range A and long‐range B compared with CTRL (Figure 3b and Table S3). These differences imply that TIS treatments remarkably affect the entire nucleus, rendering the nuclear spatial structure of these cells more disordered and generally looser. Further, the data suggest that both RAD and BLEO perturb nuclear organization, and they remodel compartmental architecture through partially distinct mechanisms.

To further investigate the distance‐dependent changes in *cis* interactions, we categorized genomic distances into 3 groups based on the RCP analysis: less than 10 Mb, 10–30 Mb, and greater than 30 Mb (Figure 3c). After dissecting compartments based on these more refined genomic distance layers, we found that compared to CTRL, both RAD and BLEO displayed different patterns in A and B compartments. Consistent with previous observations, RAD and BLEO both exhibited a loss of A compartment interactions across all genomic distances. BLEO, however, appeared to gain more B compartment interactions than RAD, particularly in extra‐long‐range (> 30 Mb) B–B interactions (Figures 3c and S4c). When we quantified compartment strength across these distance ranges, we found that, with the exception of the B compartment at genomic distances > 30 Mb, compartmentalization in both A and B compartments was consistently and significantly weaker in RAD and BLEO cells than in non‐senescent controls across genomic distances. Notably, B‐compartment strength was significantly higher in BLEO than in RAD across all distance ranges, whereas A‐compartment strength at < 10 Mb was lower in BLEO than in RAD (Figure 3d and Table S4). Collectively, these results indicate that distinct treatments elicit divergent modes of higher‐order chromatin reorganization, highlighting substantial heterogeneity in 3D genome remodeling during therapy‐induced senescence.

In aggregate, experimental data suggest that an outstanding epigenomic feature of senescent cells is the relative loss of short‐range contacts, accompanied by the simultaneous gain of long‐range contacts. Data from alternative studies involving Hi‐C experiments performed with senescent cells generated by RS and OIS are largely consistent with our findings (Olan et al. 2020; Iwasaki et al. 2019; Sati et al. 2020), supporting that these alterations are generally reproducible between different types of cellular senescence, including changes of contact probabilities, A/B compartment profiles, and interaction gain or loss.

### 2.4 Divergent Remodeling of Compartment Strength in Distinct Senescence Types

In addition to TIS, we further explored distance‐dependent 3D genome reorganization during senescence by examining the compartment strength in both OIS, RS, and deep RS models (Olan et al. 2020; Criscione et al. 2016; Sati et al. 2020). Our analysis of the relationship between contact probability and genomic distance uncovered a shared 3D genome feature of senescent cells. Compared with growing cells, both OIS and RS were characterized by reduced short‐ and mid‐range interactions accompanied by increased long‐range interactions, whereas deepRS displayed the opposite trend (Figure S5a). In contrast, overall compartment strength differed markedly among these senescence models. Relative to growing cells, RS and deepRS showed a significant reduction in global compartmentalization, whereas OIS exhibited a significant increase (Figure S5b,c). Together, these findings indicate that the nature of the senescence‐inducing stimulus dictates the specific pathway of higher‐order chromatin reorganization.

To further dissect these differences, we quantified compartment strength for short‐range and long‐range interactions within A and B compartments (Figure S5d and Table S5). A key distinction emerged, and in OIS, the long‐range B compartment strength was significantly greater than that of the A compartment. In RS and deepRS, the short‐range compartment strength is weaker than the long‐range compartment strength in both A and B compartments. Analysis of distance‐dependent compartment strength for A and B compartments provided additional resolution (Figure S5e and Table S6). In OIS, senescent cells demonstrated enhanced B compartment strength across almost the entire spectrum of genomic distances. RS and deepRS, in contrast, were characterized by a global reduction in B compartment strength. However, in the B compartment at genomic distances > 10 Mb, RS and deepRS diverged: deepRS exhibited a pattern resembling that of OIS. In TIS, BLEO showed a significantly stronger B compartment strength than CTRL at genomic distances greater than 30 Mb, a trend similar to that observed in OIS and OIS (Figures 3c,d and S5e). It is noteworthy that, regardless of the senescence type or genomic distance, the strength of B compartments generally exceeds that of A compartments, and both decrease progressively with increasing genomic distance.

In summary, our results reveal that while the gain of long‐range interactions is a general feature of 3D genome reorganization in senescent cells, the magnitude and direction of compartment strength changes differ substantially among TIS, OIS, RS, and deepRS. Specifically, OIS is uniquely characterized by a genome‐wide enhancement of B compartment strength, whereas RS results in a global attenuation of B compartment interactions; also, deepRS and RS are further distinguished in long‐distance range B compartments. These observations underscore the context‐dependent and heterogeneous nature of 3D genome remodeling during cellular senescence, shaped by the specific senescence‐inducing stimulus and the spatial scale of chromatin interactions.

### 2.5 TIS Induces TAD Instability and Structural Reorganization

**FIGURE 4:**
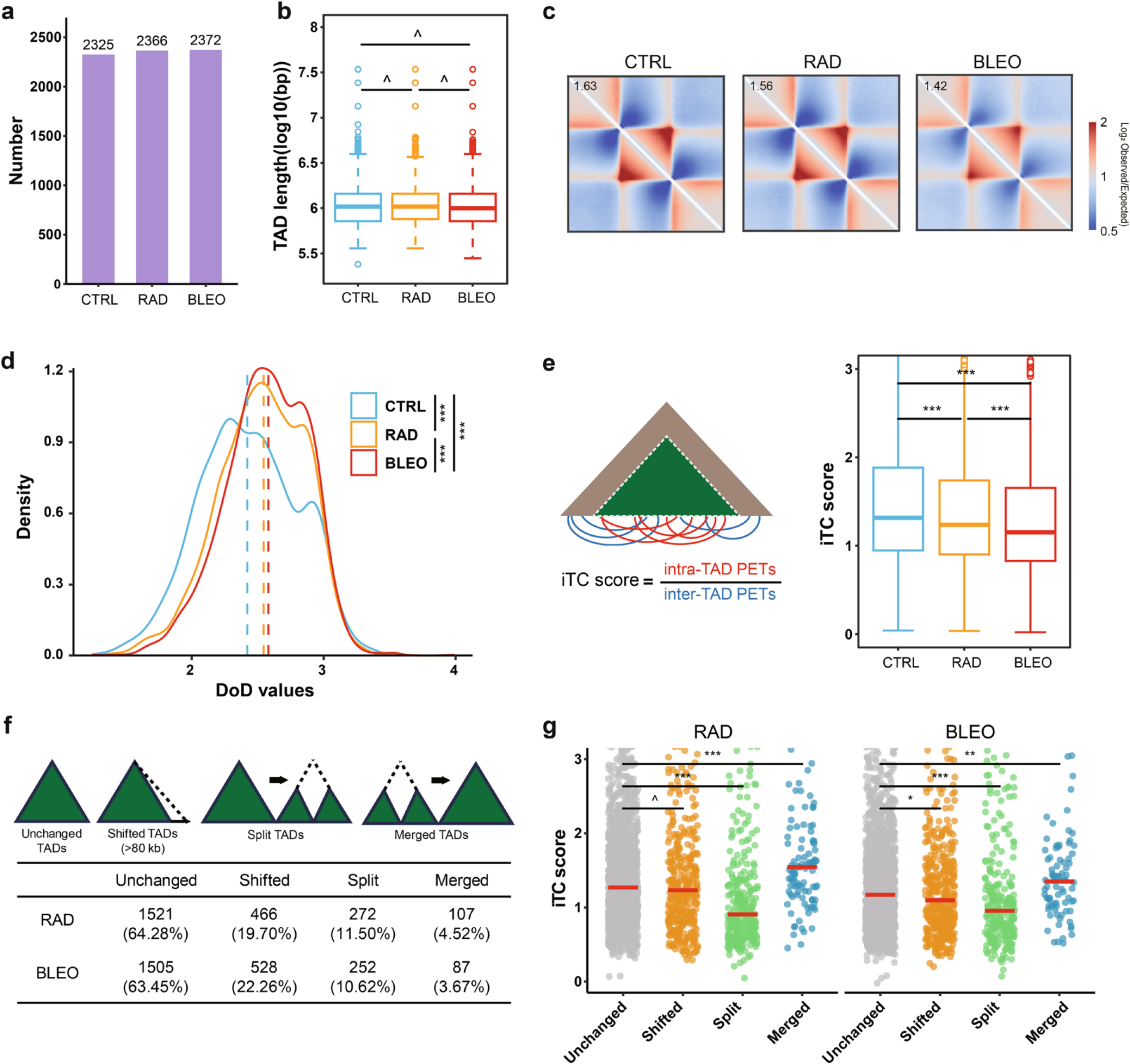
TIS induces TAD instability and rearrangement. (a) The number of TADs per sample. (b) Boxplot presentation of TAD length. (c) Shown are rescaled observed/expected pileup heatmaps of normalized Hi‐C contact enrichment centered on TADs at 40 kb resolution in CTRL, RAD, and BLEO. Colors indicate contact enrichment (scale bar), and numbers denote the aggregate enrichment score for each condition. (d) Density distribution of degree of disorder (DoD) values in CTRL, RAD, and BLEO, respectively. The dashed line indicates the median of the DoD value of each sample. (e) Diagram of calculating intra‐TAD connectivity (iTC) score (right). Boxplot of TAD connectivity score for each sample (left). (f) TAD rearrangement events and the statistics of different types of rearrangement events for RAD and BLEO compared to CTRL during TIS. (g) Dot plot showing TAD connectivity score for different rearrangement events of RAD and BLEO. The *p* values were calculated by unpaired Wilcoxon tests. ^*p* > 0.05; *p < 0.05; **p < 0.01; ***p < 0.001.

Next, we continued to examine the changes in topologically associated domains (TADs) during TIS. We identified 2325, 2366, and 2372 TADs in CTRL, RAD, and BLEO conditions, respectively, with no significant differences in their lengths (Figure 4a,b). This suggests that the overall TAD architecture is largely preserved during cellular senescence. To further explore intra‐TAD interactions, we performed aggregate TAD analysis (ATA), where TADs were rescaled to a uniform dimension to enable direct comparisons across different treatments. As cellular senescence induced global chromatin decompaction, the frequency of chromatin interactions was reduced within senescent cells, accompanied by a weakening of insulation at TAD boundaries (Figures 4c and S6a). Senescence can be conceptualized as a process of increasing entropy, reflected in the global relaxation of chromatin organization and a reduced degree of chromatin folding within TADs (Liu et al. 2022). To quantitatively assess the stability of TADs and thereby capture their structural states, we calculated relevant metrics. Specifically, we derived the consolidation score for each TAD, with the consolidation score consistently lower in senescent cells compared to non‐senescent controls, indicating weaker TAD stability (Figure S6b and Table S7). To further quantify the organizational integrity of TADs, we calculated the degree of disorder (DoD) for each TAD by measuring the average distance between important interactions within the TAD (Lin et al. 2022). Quantitative analysis revealed that the DoD values of TADs were significantly higher in senescent cells under RAD and BLEO treatment compared to CTRL, and 65.72% of TADs in RAD and 74.27% in BLEO exhibited increased disorder relative to CTRL, suggesting that TADs in these senescent states exhibit increased disorganization (Figure 4d and Table S8). These findings underscore that TAD‐internal interactions become destabilized during TIS.

To capture the degree of structural compartmentalization of TADs, we used the intra‐TAD connectivity (iTC) score, which was defined as the ratio of the pair‐end tags (PETs) within one TAD to that between adjacent TADs (Ma et al. 2024). Alterations in iTC scores provide insights into potential changes in 3D genome organization under distinct cellular conditions. Overall, the iTC score was reduced during TIS (Figure 4e). We employed the iTC score to identify differential TADs. Compared to CTRL, we identified 250 and 209 TADs with significantly increased iTC scores in RAD and BLEO, respectively, and 318 and 323 TADs with significantly decreased iTC scores in these same conditions (Figure S6c,d and Table S9). Compared with TADs that showed no significant changes in connectivity, the differential TADs were predominantly located within the active A compartment and exhibited stronger boundary insulation, higher chromatin accessibility, and increased CTCF density. With the exception of PC1 values, the other average signal density profiles of these differential TADs had modest differences between CTRL and senescent cells. Notably, in the increased connectivity TADs of RAD and BLEO, we detected upregulated expression of the pregnancy‐specific beta‐1 glycoprotein (*PSG*) gene family, a finding consistent with observations in senescent human mesenchymal progenitor cells (hMPCs) (Liu et al. 2022). During TIS, *PSG* genes also underwent a compartmental switch from B to A compartment. This was accompanied by an increase in local interaction frequencies within the *PSG*‐containing TAD, which likely contributed to the cooperative activation of *PSG* genes (Figure S6f). This exemplifies a typical scenario in which loose chromatin during senescence facilitates the release of transcriptional repression and drives gene activation in senescent cells.

In addition to changes in interaction strength, we also sought to investigate the repositioning of TADs as regulatory units within the genome during TIS. Consequently, we examined TAD rearrangements that occurred during TIS. Relative to CTRL, approximately 64% of TADs remained unchanged in RAD and BLEO conditions, while about 20% exhibited boundary shifts and 11% underwent split events. The remaining TADs experienced merged events (Figure 4f). Notably, TADs that underwent merge or boundary shift were longer on average compared with unchanged TADs, whereas split TADs tended to be shorter (Figure S6g). In terms of connectivity, merged TADs exhibited the highest connectivity scores, while split TADs showed the lowest (Figure 4g).

These results indicate that changes in TAD rewiring during TIS are not uniformly distributed, but instead preferentially occur within active genomic regions, which reflects a selective reorganization of chromatin interactions in these critical domains upon TIS.

### 2.6 TIS Induces Extensive Loss of Chromatin Loops

**FIGURE 5:**
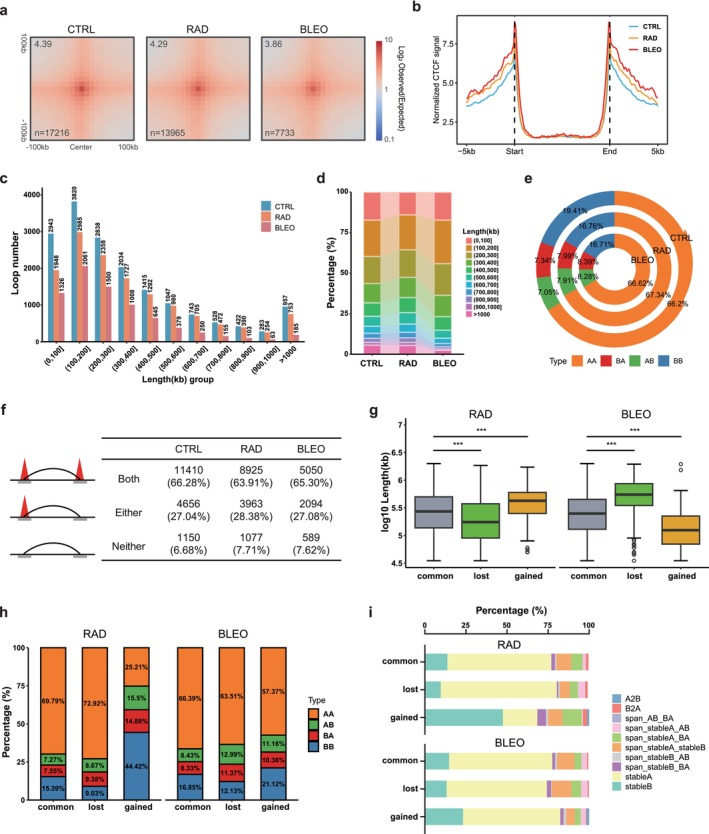
RAD and BLEO cause loop rewiring in different ways. (a) The APA plots for all loops confirm strong local Hi‐C contact enrichment. The top right corner of the heatmap shows the value of the center pixel, and the bottom left corner shows the number of loops. (b) Profile for CTCF density around loops. (c) Length distribution of loops of each sample. (d) The proportion of different loop length groups. (e) The proportion of loops in different compartment types. (f) Diagram of the CTCF binding sites at loop anchors. (g) Length comparison of differential loops. The *p* values derived from the unpaired Wilcoxon test, (h) The proportion of differential loops in different compartment types. (i) The proportion of differential loops in different types of compartment‐switched regions.

High‐resolution Hi‐C maps revealed prominent regions of high interaction frequencies. Using HiCCUPS (Rao et al. 2014) at 5 kb and 10 kb resolutions, we identified 17,216, 13,965, and 7733 loops in CTRL, RAD, and BLEO conditions, respectively. The aggregate peak analysis (APA) over all loops confirmed strong local Hi‐C contact enrichment (Figure 5a). As CTCF often acts as an architectural protein defining TAD and loop boundaries, the profile of CTCF density around loop anchors in all three conditions showed pronounced CTCF enrichment (Figure 5b). Compared with CTRL, we identified 1622 and 1530 CTCF binding sites with decreased occupancy and 1013 and 1812 sites with increased occupancy in RAD and BLEO, respectively. Differential CTCF peaks in RAD and BLEO were associated with distinct functional shifts, with gained sites enriched for CTCF/BORIS motifs and frequently co‐enriched for AP‐1 family motifs (Figure S8a,b).

We further conducted precise length‐based stratification of all loops. The majority of loops corresponded to short‐range interactions, with only a minority spanning long‐range distances (> 1 Mb) (Figure 5c). Specifically, loops shorter than 200 kb accounted for approximately 40%, 35%, and 45% of their whole loops in CTRL, RAD, and BLEO, respectively. Loops longer than 500 kb comprised 23.1%, 25.4%, and 14.6% of total loops in these conditions, while loops exceeding 1 Mb represented 5.5% in CTRL and RAD, but only 2.4% in BLEO (Figure 5c,d). Consistent with the observations in Figure 2c, TIS is accompanied by a pronounced reduction in short‐range interactions, which largely accounts for the observed differences in loop numbers across the 3 conditions. Since chromatin loops play essential roles in organizing local chromatin architecture, the dramatic loss of loops during senescence likely leads to significant alterations in the 3D chromatin structure and the surrounding genomic environment.

Chromatin loops, as fine‐scale structural features, are constrained within larger‐scale chromatin architecture units such as compartments and TADs (Yang et al. 2023). We therefore further investigated the spatial relationships of loops with these higher‐order structures to better understand their regulatory landscape in senescent cells. First, we classified loops based on the compartment identity of their two anchors into 4 types: AA, BB, AB, and BA (Figure S7a). Approximately 66% of loops in all 3 conditions fell into the AA type, which was also the predominant configuration across different loop lengths (Figures 5d and S7b). Notably, as loop length increased, the proportion of AA loops decreased while that of BB loops increased. Next, we examined the spatial localization of loops relative to compartment switch regions. In both RAD and BLEO cells, ~62% of loops were located in Stable‐A regions, while ~15% were located in Stable‐B regions (Figure S7c,d). These findings indicate that loops preferentially localize within the transcriptionally active and more accessible A compartments, in contrast to the more compact and repressive B compartments. Moreover, shorter loops were more likely to be located within active chromatin regions, possibly reflecting their greater spatial flexibility. In contrast, longer loops, which require overcoming additional structural constraints, might engage more complex interaction networks, potentially playing a role in the spatial regulation of specific genes. Such spatial stratification provides an ordered structural framework for gene regulation and expression. Finally, we analyzed the relationship between loops and TADs and found that in CTRL and BLEO cells, approximately 90% of loops were confined within a single TAD (Figure S7e,f). This suggests that stable loops are positioned to preserve local chromatin interactions within an entire TAD, potentially safeguarding gene expression patterns from aberrant spatial contacts.

### 2.7 Inducer‐Specific Dynamics and Distinct Patterns of Differential Loops During TIS

We compared the loops identified in RAD and BLEO with those in CTRL, categorizing them into 3 groups: common, lost, and gained. Employing cLoops2 with stringent parameter settings, we detected 564 lost loops and 478 gained loops in RAD, and 1052 lost loops and 252 gained loops in BLEO (Figure S8c,d and Tables S10, S11). To explore the relationship of CTCF binding sites and loops, we first found that the majority of loops displayed CTCF binding at both anchors, with ~27% of loops showing CTCF binding only at one single anchor (Figure 5f). We then focused on differential CTCF sites and mapped them onto loops (Figure S8e). Differential CTCF peaks are scattered along the chromosomes without obvious positional bias; only 14.6% (RAD) and 14.9% (BLEO) of loops carry a differential CTCF peak at both anchors, and 22.4% (RAD) and 27.1% (BLEO) contain a differential CTCF peak somewhere inside the loop domain (Figure S8f). When restricting the analysis to differential loops, 12.8% and 16.7% of RAD‐gained and RAD‐lost loops, and 13.5% and 18.7% of BLEO‐gained and BLEO‐lost loops, respectively, have at least one anchor with a differential CTCF peak, suggesting RAD and BLEO were similar in these dynamic loop behaviors (Figure S8g). Thus, the majority of weakened or lost loops do not coincide with local gain or loss of CTCF. This observation suggests that the loop loss observed during TIS may not be primarily driven by gain or loss of CTCF binding sites.

We next explored the patterns of differential loops. Interestingly, the length distributions of these differential loops exhibited opposite trends between RAD and BLEO (Figure 5g). RAD lost approximately 56% of its loops shorter than 200 kb and gained around 28% of loops longer than 500 kb. In contrast, BLEO lost 52% of its loops longer than 500 kb and gained 72% of loops shorter than 200 kb (Figure S8h). Similarly, RAD lost more AA‐type loops while gaining 44.42% of BB‐type loops. The lost and gained loops of BLEO predominantly consisted of AA‐type loops, with only 21.12% of BB‐type loops gained (Figure 5h). Regarding the spatial localization of differential loops in relation to compartment switch regions, RAD gained 47.5% of Stable‐B loops, whereas BLEO gained only 23.2% of Stable‐B loops (Figure 5i). Analysis of CTCF binding sites near differential loops revealed that most gained loops in both RAD and BLEO were either anchored at a single CTCF site or lacked CTCF at both anchors (Figure S8i), suggesting that the newly formed loops during TIS might be less structurally stable. We found that the average ATAC‐seq and CTCF signal intensities surrounding the gained loops in RAD were lower than those of its common and lost loops. In contrast, the gained loops in BLEO were associated with higher average ATAC‐seq and CTCF signal intensities compared to their common and lost loops (Figure S8j,k). These findings underscore that different TIS inducers drive divergent loop remodeling patterns and highlight a potential compromise in the architectural stability of chromatin loops formed under senescence conditions.

Senescence‐induced chromatin remodeling creates new opportunities for *cis* regulatory elements to interact with their target genes, with enhancer–promoter (E‐P) loops playing a crucial role in gene regulation (Liu et al. 2022; Zhao et al. 2023). We categorized and quantified loops based on the spatial relationship between enhancers and promoters, finding that E‐P loops accounted for 18%, 37%, and 32% of all loops in CTRL, RAD, and BLEO, respectively. Of note, both RAD and BLEO lost and gained the largest proportions of E‐P loops, while also gaining a substantial fraction of NN‐type structural loops (Figure S8l,m). Upon TIS, we observed genes associated with BLEO‐gained loops show slightly higher expression than those at BLEO‐lost loops (Figure S9b), but we do not detect a clear relationship between the magnitude of loop‐strength changes and gene‐expression changes at the individual gene level (Figure S9a,b). Both RAD and BLEO lost E‐P loops related to genes involved in DNA replication, cell cycle progression, and cell proliferation. In contrast, the gained E‐P loops were linked to genes involved in nucleotide biosynthesis, protein degradation regulation, and signaling pathways (Figure S9c–e). These findings suggest that while RAD and BLEO share similar biological processes affected by differential E‐P loops, the direct coupling between E‐P loop remodeling and gene expression is uniquely enhanced in BLEO, underscoring therapy‐specific modes of 3D genome reorganization during senescence.

### 2.8 Spatial Decondensation and SASP Gene Activation During TIS

**FIGURE 6:**
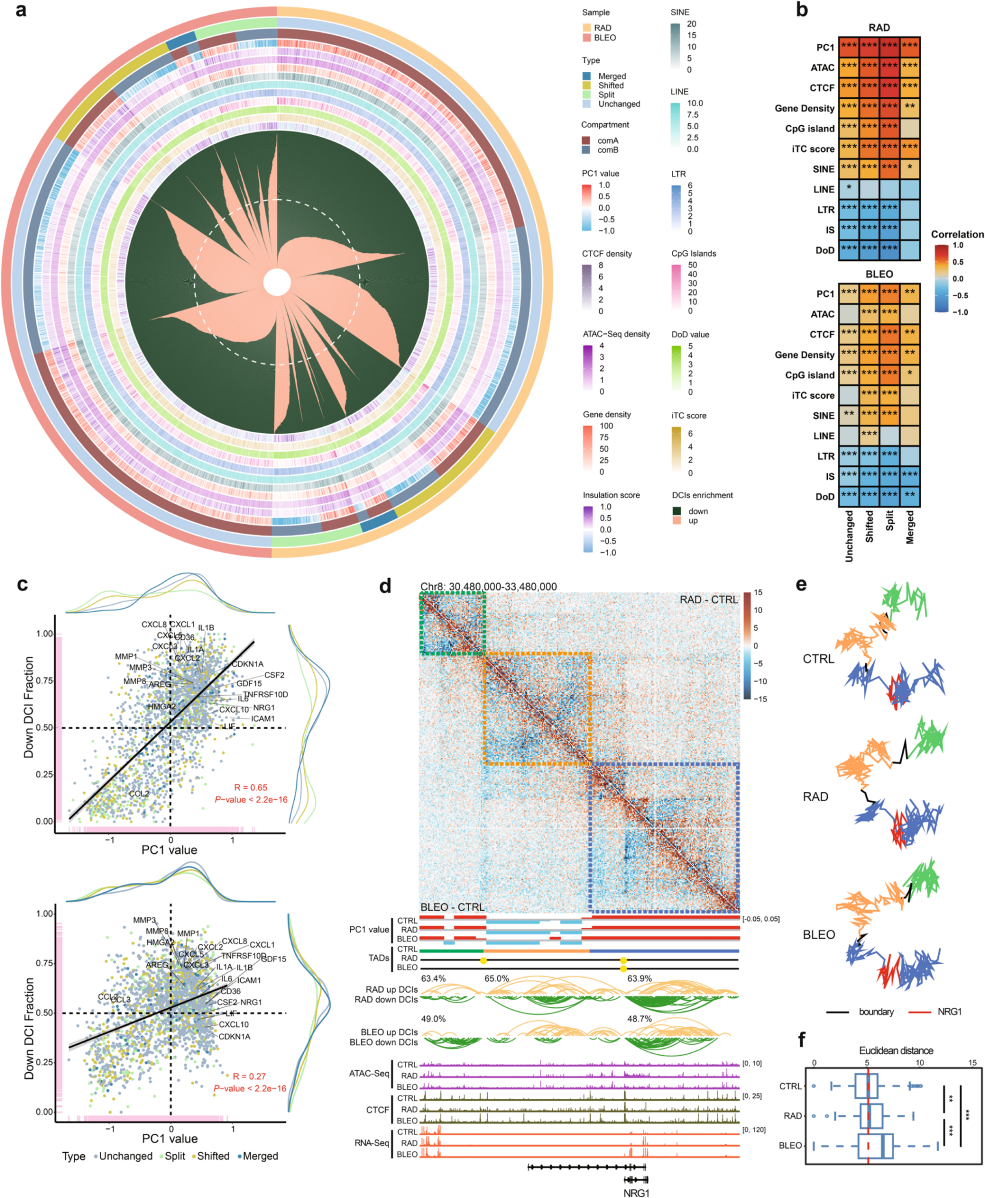
TIS provides moderate spatial reorganizations for SASP factors. (a) For each TAD genome‐wide, the circle plot shows the average signal of multiple genomic features (e.g., PC1, CTCF CUT&RUN, ATAC‐seq, gene and repeat density, CpG islands, insulation score, and DoD scores) and visualized them as thin concentric bars around the circle. The central petal plot summarizes, for RAD and BLEO and separately for A and B compartments, the per‐TAD fractions of up‐ and downregulated differential chromatin interactions (DCIs) across classes of TAD reorganization; each petal comprises thousands of bars (one per TAD) ranked by decreasing downregulated DCI fraction (white dashed line, 0.5). (b) The heatmap shows the Spearman correlation coefficients of the proportion of downregulated chromatin interactions in each TAD and other indicators calculated according to sample and TAD rearrangement type. The *p* value is marked in the box, *p < 0.05; **p < 0.01; ***p < 0.001. (c) The scatter plot shows the Spearman correlation coefficient and *p* value of the proportion of downregulated interactions and PC1 values according to sample and TAD rearrangement type. The upregulated SASP factors in RAD or BLEO are also marked. (d) PC1 values, TADs, differential chromatin interactions, and epigenetic landscapes around the *NRG1* locus. TADs of CTRL are marked with different colors and displayed as boxes in dotted lines on the differential heatmap, while TAD boundaries of RAD and BLEO are represented by yellow circles. For RAD and BLEO, the fraction of down‐regulated DCIs is annotated next to the interaction arcs in the genome tracks for each corresponding TAD. (e) 3D modeling predictions are performed according to the genome range and CTRL TADs in d. Lines of different colors represent CTRL TADs in d, black lines are TAD boundaries, and red lines are the location of *NRG1*. (f) Euclidean distance between the location of *NRG1* and other locations in the TAD represented by the blue line segment. The *p* values were calculated by paired Wilcoxon tests, *p < 0.05; **p < 0.01; ***p < 0.001.

We explored TAD rearrangements, which can represent notable reorganizations of higher‐order chromatin architecture and spatial positioning as the structural units to investigate differential chromatin interactions. These events are often associated with the redistribution of functional genomic regions (Kloetgen et al. 2020). Consequently, focusing on TAD rearrangements provides a more comprehensive understanding of how large‐scale structural alterations contribute to changes in chromatin interactions and gene regulation. In senescent cells, we first mapped TAD rearrangement events to their compartment locations and quantified the proportion of upregulated and downregulated differential chromatin interactions (DCIs) within each TAD. We also calculated the enrichment of various signals within individual TADs. In the context of TIS, regardless of the type of TAD rearrangement event, TADs located within the A compartment displayed a higher proportion of downregulated DCIs, while TADs in the B compartment predominantly contained upregulated DCIs (Figures 6a and S10a,b, Table S12). Notably, the downregulated DCIs showed positive correlations with features associated with open chromatin environments, including higher PC1 values, ATAC‐seq, CTCF, gene density, CpG island density, and the presence of short interspersed nuclear elements (SINEs) (Figures 6b and S10c). This suggests that downregulated DCIs are a hallmark of more accessible chromatin regions.

To elucidate the relationship between TAD rearrangement events and downregulated DCIs, we applied two complementary approaches. In the RAD condition, compared with unchanged TADs, the proportions of downregulated DCIs differed significantly among TAD rearrangement types, with split TADs exhibiting the lowest proportion of downregulated DCIs and merged TADs exhibiting the highest. In contrast, in the BLEO condition, the proportion of downregulated DCIs had slight changes across the various TAD rearrangement types (Figure S10d). To further quantify the likelihood of each TAD rearrangement type contributing to high downregulated DCI fractions compared with unchanged TAD, we employed logistic regression analysis. In the RAD condition, shifted and split TADs showed a significantly reduced log odds ratio (logOR), indicating a lower propensity to reach high downregulated DCI fractions, whereas merged TADs showed a small, nonsignificant increase. In the BLEO, all types exhibited a modest but not significant decrease in logOR compared to unchanged TADs (Figure S10e). These findings suggest that TAD rearrangement events are associated with a reduced propensity to exhibit high fractions of downregulated DCIs in RAD‐induced senescence, whereas such a trend appears less pronounced in BLEO‐induced conditions.

Furthermore, we observed that many SASP factors are located in regions characterized by a high fraction of downregulated DCIs. Among them is *NRG1*, previously reported to be regulated by 3D genome architecture in the OIS model of human IMR90 cells (Sati et al. 2020), which has been implicated in cellular senescence through its role in modulating cell survival, inflammation, and tissue repair responses (Olan et al. 2020). In both RAD and BLEO conditions, *NRG1* resides within a shifted TAD and exhibits a notable increase in downregulated DCIs near its promoter region, accompanied by enhanced ATAC‐seq accessibility in RAD (Figure 6d). Additionally, 3D structural modeling of the NRG1 locus indicates that TIS promotes a more open chromatin environment in this region (Figure 6e,f). *HMGA2* is essential for the formation of senescence‐associated heterochromatin foci (SAHF), as it facilitates chromatin condensation and structural reorganization necessary for establishing stable SAHF structures (Sati et al. 2020; Olan et al. 2023). *HMGA2*, which shows specific upregulation in BLEO, also displays a substantial number of downregulated DCIs around its promoter in this condition (Figure S11a). Similarly, members of the MMP and CXCL gene families are upregulated in both RAD and BLEO conditions, likely driven by chromatin structural reorganization (Figure S11b–e). Through extracellular matrix remodeling and immune cell recruitment, MMPs and CXCL chemokines synergistically amplify the senescence‐associated secretory phenotype and its pro‐inflammatory effects (Wang et al. 2022). Although *CCL2* resides in regions with a low proportion of downregulated DCIs and within the B compartment, it is still highly expressed in both RAD and BLEO conditions, accompanied by increased ATAC‐seq accessibility compared with CTRL (Figure S12a,b). *LIF* is not located in regions with a markedly high proportion of downregulated DCIs, yet it resides within the A compartment and is enriched with ATAC‐seq signals, showing elevated expression during TIS (Figure S12c,d). Collectively, these findings suggest that during TIS, the chromatin environment surrounding SASP genes generally becomes more accessible and decondensed, as evidenced by increased active signals and redistribution of downregulated DCIs. The expression of SASP factors remains highly heterogeneous, reflecting that 3D genome remodeling modulates their expression in diverse ways rather than uniformly in a single manner.

## 3 Discussion

**FIGURE 7:**
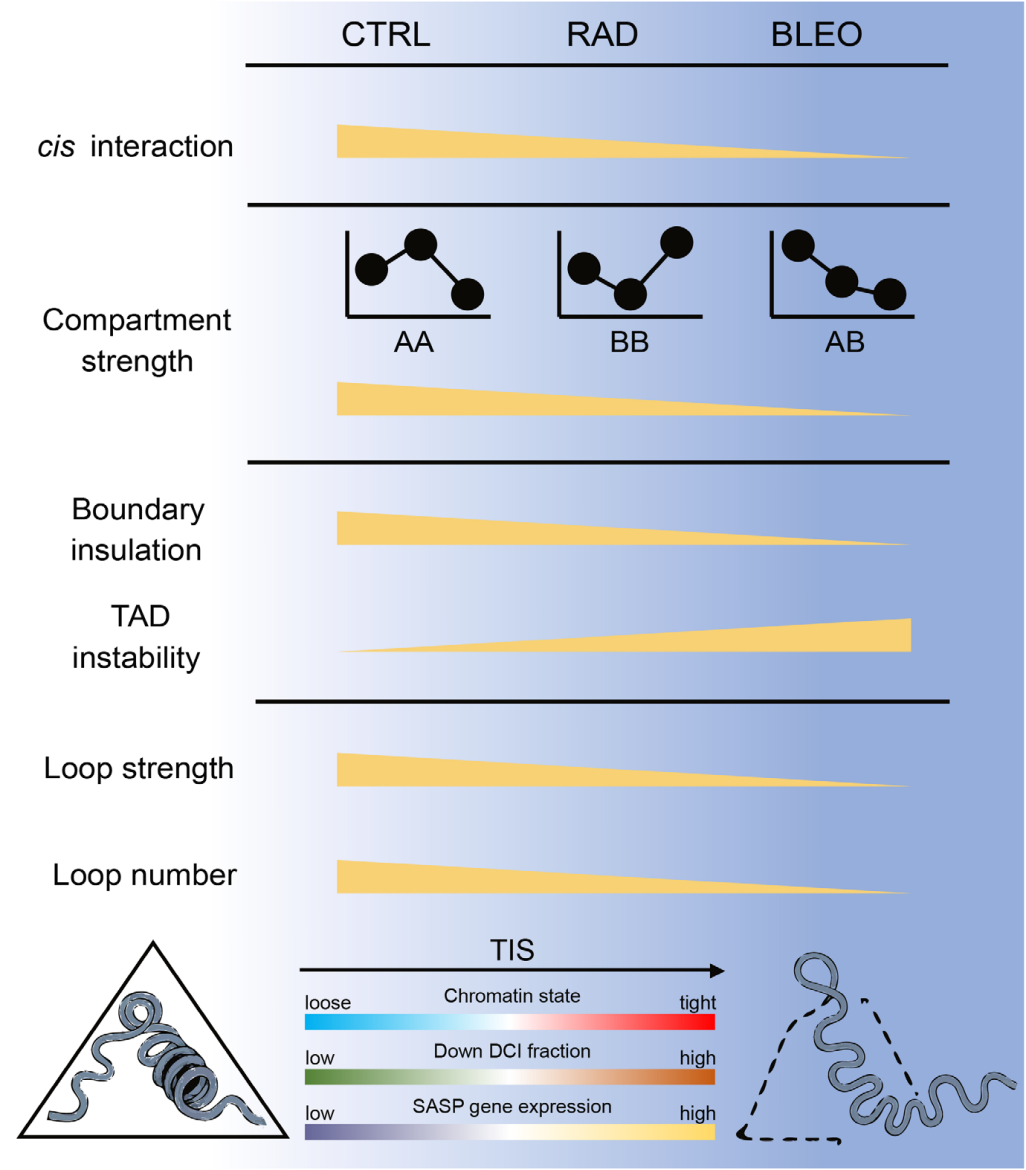
Schematic diagram of 3D genome remodeling and spatial changes around SASP factors during TIS. During TIS, chromatin architecture undergoes multilayered reorganization, characterized by a progressive decline in *cis* interaction frequency, compartment strength, boundary insulation, loop strength, and loop number, accompanied by TAD instability, collectively reflecting an entropy‐increasing process. The extent and direction of these structural alterations vary depending on the senescence‐inducing stimulus. Notably, regions surrounding SASP factors exhibit loosened chromatin states, increased fraction of downregulated DCIs, and elevated SASP expression, highlighting a spatial regulatory shift that favors the development of a senescence‐associated phenotype and formation of an inflammatory microenvironment.

The genome‐wide reorganization of 3D chromatin architecture during TIS, a form of stress‐induced premature senescence (SIPS), and its direct impact on global transcription remain poorly characterized. To address this, we integrated Hi‐C, ATAC‐seq, CUT&RUN, and RNA‐seq to map the multidimensional chromatin landscape in TIS. We found that TIS triggers global chromatin decondensation, destabilizes TADs, and induces context‐specific remodeling of compartments, chromatin loops, and interactions. Collectively, these alterations establish a permissive chromatin conformation that facilitates the activation of SASP gene expression (Figure 7). We observed that the extent and nature of these 3D architecture changes vary depending on the senescence‐inducing agent, highlighting the heterogeneous chromatin responses that underline TIS and its therapy‐specific regulatory programs, which are not a monolithic state but rather a spectrum of conditions shaped by the initial DNA insult.

A critical driver of 3D genome reorganization in cellular senescence is the dysregulation of heterochromatin (Zhang et al. 2021). Both RAD and BLEO treatments lead to a notable decrease in overall compartmentalization strength, indicative of a profound disruption in chromatin higher‐order structure. This aligns with the broader understanding of heterochromatin loss, such as the depletion of repressive histone marks like H3K9me3 and the detachment of chromatin from the nuclear lamina, as a hallmark of aging and senescence (Liu et al. 2022). Unlike the reported OIS models in IMR90 that show extensive compartment switching (Iwasaki et al. 2019), TIS in HDFs exhibits a relatively modest proportion of A/B compartment conversions. More broadly, the fraction of the genome undergoing compartment conversion is often modest across multiple senescence contexts, underscoring substantial heterogeneity in how distinct senescence programs remodel compartment identity (Liu et al. 2022; Guan et al. 2020; Dalgarno et al. 2025; Zhang et al. 2021). However, the nature of intra‐compartment interaction changes is inducer‐specific: RAD treatment significantly reduces heterochromatin–heterochromatin interactions, whereas BLEO treatment, despite overall weakening, tends to promote B–B interactions at very long ranges. These changes, coupled with a relative loss of short‐range contacts and a gain in long‐range contacts, suggest a general decondensation and increased disorder of the chromatin landscape in TIS. The differences in senescence inducer, cell type, time point, and analysis strategy all likely contribute to the differences between compartment switching observed in our study and compartment switching reported in other senescence models.

When compared to other forms of cellular senescence, TIS presents both shared and unique 3D genomic features. Like OIS and RS, TIS is characterized by a decrease in short‐ and medium‐range chromatin interactions and an increase in long‐range interactions, suggesting a more spatially expanded chromatin configuration. However, overall compartment strength diverges: TIS generally shows a reduction, contrasting with a significant increase in OIS and a marked reduction in RS. Notably, BLEO‐induced TIS shows stronger B‐compartment strength at genomic distances greater than 30 Mb, a feature somewhat similar to OIS and deep RS, whereas RAD was more similar to the globally weakened architecture seen in RS. The increased long‐range B–B interactions in BLEO–TIS might represent a different, perhaps less condensed, form of heterochromatin aggregation. SAHFs, a hallmark of OIS, do not explicitly emerge in the reported RS and TIS models (Sati et al. 2020). It is worth noting that, despite sharing many characteristics with replicative senescence, TIS is induced on a markedly different timescale. While replicative senescence in human cells typically takes multiple population doublings to develop in vitro, TIS can be triggered rapidly following brief exposure to anticancer agents or radiation (López et al. 2025; Pacifico et al. 2024). These distinctions underscore that different senescence‐inducing stimuli sculpt unique 3D genome architectures, likely contributing to the observed heterogeneity in senescent phenotypes, including composition of the SASP.

Beyond general decompaction, we observed marked TAD instability during TIS, accompanying reduced insulation at TAD boundaries. Although the number and length of TADs remained largely unchanged, quantitative metrics such as intra‐TAD connectivity, consolidation score, and degree of disorder consistently indicated weakened architectural integrity. During RS, local chromatin interactions within the TAD encompassing the PSG gene family that is upregulated in senescent hMPCs undergo rewiring, which weakens association with the nuclear lamina and creates a more relaxed chromatin state (Liu et al. 2022). The expression of the PSG gene family is upregulated during TIS, accompanied by enhanced intra‐TAD interactions and a compartment switch from B to A. This highlights how chromatin reorganization facilitates gene activation. These results support the model that TAD instability reflects increased structural entropy during senescence, enhancing the plasticity of gene regulation in response to stress.

Loop‐level analysis reveals that TIS leads to widespread loss of chromatin loops, particularly those spanning short genomic distances below 500 kb. The patterns of lost and gained loops are inducer‐specific. RAD treatment is characterized by the loss of short‐range loops and the gain of long‐range BB‐type loops, which are associated with relatively low ATAC‐seq and CTCF signals, suggesting reduced chromatin accessibility and architectural support. In contrast, BLEO treatment leads to the loss of long‐range loops and a gain of short‐range AA‐type loops enriched in accessible chromatin. These distinct remodeling patterns extend to E‐P loops: both RAD and BLEO show a general loss of E‐P loops linked to proliferation genes and a gain of those associated with signaling pathways. In BLEO, E‐P loop strength positively correlated with gene expression changes, suggesting functionally relevant rewiring, whereas RAD E‐P loops lacked such a relationship. This may underline the more robust transcriptional activation of SASP genes in BLEO‐treated cells compared with RAD.

The 3D genome alterations in TIS play a pivotal role in regulating SASP gene expression through TAD rearrangements and DCIs. Structural changes such as shifted, split, and merged TADs disrupt the local regulatory landscapes (Kloetgen et al. 2020). A notable observation is that TADs within the A compartment tend to exhibit more downregulated DCIs, while those in the B compartment show an increase in upregulated DCIs. Interestingly, these downregulated DCIs are paradoxically enriched in features of open and active chromatin, such as high PC1 values, increased chromatin accessibility, and dense CTCF binding, suggesting that the loss of specific, possibly restrictive, chromatin contacts may promote SASP gene activation. This chromatin decompaction and spatial reorganization likely facilitate regulatory rewiring, enhancing the transcriptional activity of inflammation‐related loci during senescence. *HMGA2* is a chromatin‐associated architectural protein that promotes the formation of SAHF, a hallmark feature observed predominantly in OIS (Sati et al. 2020). In our study, *HMGA2* was specifically upregulated in BLEO‐induced senescence, but not in the RAD condition. Notably, this upregulation was accompanied by a substantial increase in the number of downregulated DCIs in the vicinity of the *HMGA2* locus in BLEO cells, a pattern not observed under RAD treatment. This structural reorganization, together with enhanced B compartment strength at long genomic distances above 30 Mb, mirrors features previously reported in OIS models, suggesting that BLEO may partially recapitulate OIS‐like chromatin patterns. These observations further highlight the therapy‐dependent variability in senescence‐associated 3D genome remodeling. Lastly, we show that chromatin remodeling around SASP genes is heterogeneous yet functionally consequential. Genes such as *NRG1*, *MMP*, and the *CXCL* gene family resided within regions of highly downregulated DCIs, while others, such as *CCL2* and *LIF*, were upregulated without major topological changes, reflecting diverse regulatory strategies. This heterogeneity suggests that 3D genome remodeling acts as a modulatory rather than deterministic force in SASP gene regulation, enabling selective activation within a permissive but structurally varied chromatin context.

The strength of this study is the direct comparison of two typical TIS inducers under experimental conditions, substantiating that TIS triggers complex and therapy‐specific remodeling of the 3D genome. Radiation and bleomycin are intrinsically genotoxic, and we therefore acknowledge that residual DNA lesions and heterogeneous repair states could, in principle, influence population‐averaged chromatin contact maps and complicate the interpretation of loop‐ and compartment‐level differences. Locus‐specific validation, such as DNA‐FISH and orthogonal mapping of lamina association via lamin ChIP‐seq or CUT&RUN, would provide valuable independent support for our conclusions. In particular, targeted validation of 3D architectural changes at selected SASP loci in TIS is warranted to confirm regional remodeling inferred from population‐averaged measurements. By integrating Hi‐C, ATAC‐seq, and transcriptomic data, we provide a layered view of genome regulation during TIS. We further note that TIS is not a monolithic state and that, under our conditions, only a fraction of the population enters a fully senescent program. This incomplete penetrance is expected to attenuate effect sizes and dilute‐like senescence‐associated signals in bulk assays. Moreover, bulk chromatin conformation datasets like Hi‐C and ATAC‐seq are inherently sparse and may obscure cell‐to‐cell variability, average over heterogeneous cell states (there appears to be an approximately 30%–40% non‐senescent fraction of cells), limiting our ability to resolve dynamic, cell‐to‐cell variability in 3D genome organization. Future studies using single‐cell or spatial omics approaches, combined with perturbation of architectural proteins such as CTCF or cohesin, will be needed to clarify the functional consequences of 3D genome remodeling in senescence. Finally, radiation and bleomycin differ in their mechanisms of action and cellular consequences, and our data are consistent with the view that the resulting 3D genome responses integrate both senescence‐associated remodeling and stimulus‐specific, potentially non‐senescent stress programs, which together shape the heterogeneous transcriptional outputs, including the composition and magnitude of the SASP. Overall, this work outlines the scale and complexity of chromatin reorganization during TIS and underscores its dependence on the senescence‐inducing context or environmental stimulation.

## 4 Methods

### 4.1 Cell Culture

Human primary prostate fibroblast cell line PSC27 was generously provided by Dr. Peter Nelson (Fred Hutchinson Cancer Center), grown at 37°C with 5% CO_2_, and maintained in a pre‐optimized stromal complete medium as described previously (Sun et al. 2012). Cells were serially passaged (passage number < 30) by trypsinization at 1:4 dilution after reaching > 90% confluence (CTRL), exposed to 10 Gy ionizing radiation (RAD) or 50 μg/mL bleomycin (BLEO). The doses of treatments were determined a priori. Senescence was experimentally verified by staining for senescence‐associated β‐galactosidase (SA‐β‐Gal) and 5‐bromo‐2′‐deoxyuridine (BrdU). All lines were routinely tested for mycoplasma contamination and authenticated with STR assays.

### 4.2 Immunoblot Analysis

Whole cell lysates were prepared using RIPA lysis buffer supplemented with protease/phosphatase inhibitor cocktail (Biomake). Nitrocellulose membranes were incubated overnight at 4°C with primary antibodies, and HRP‐conjugated goat anti‐mouse or anti‐rabbit served as secondary antibodies (Vazyme).

Cells were cultured as described in Supplementary Methods. Treatment for 7 days with 100 μM 5‐bromo‐2′‐deoxyuridine (BrdU) (Sigma, Catalog No. B5002) dissolved in DMSO (Sigma, Catalog No. D2650) was used to induce senescence. A 0.1% DMSO solution in the appropriate cell line growth media was used as a control. The primary antibodies employed in this study are listed in Table S13.

### 4.3 RNA Library Preparation and RNA‐Seq Data Processing

Cells were rinsed with PBS once before being lysed on a plate with TRIzol (Magen). The lysates were transferred to the DNase–RNase‐free tubes and incubated at room temperature for 5 min. Thereafter, samples were vortexed for 20 s before 0.2× volumes of chloroform were added, followed by tube inverting/mixing and sample centrifugation at 13,000 rpm at 4°C for 15 min. The aqueous phase was then processed using the RNeasy Mini Kit (QIAGEN) with DNase treatment, according to the manufacturer's instructions. Purified RNA (minimum of 1 μg) was used for library preparation with the TruSeq Stranded Total RNA Library Kit according to the manufacturer's instructions.

RNA‐seq reads were mapped to the hg19 reference genome using Hisat2 (version 2.2.1). The aligned reads were calculated for each protein‐coding gene using HTSeq (version 2.0.2) with default parameters. Fragments Per Kilobase of exon model per Million mapped fragments (FPKM) were calculated by StringTie (version 2.2.1). The differentially expressed genes (DEGs) with adjusted *p* value < 0.01 and absolute log_2_(fold change) > 1 were identified by DESeq2 (version 1.34.0). Gene ontology was conducted with the online enrichment analysis tool DAVID.

### 4.4 RNA Extraction and Real‐Time Quantitative PCR

Total RNA samples were extracted from cultured cells and tissues using TRIzol (Magen). RNA was reverse transcribed to cDNA using TransScript First‐Strand cDNA Synthesis SuperMix (Tiangen, KR118‐02) following the manufacturer's instructions. RT‐qPCR was performed using Hieff qPCR SYBR Green Master Mix (YEASEN, 11202ES08) on an ABI QuantStudio7 Flex (ABI Q7). Data were analyzed using the 2(−ΔΔCt) method. RPL13A or GAPDH was used as a control to normalize the expression of target genes.

### 4.5 ATAC‐Seq Protocol

Cells for each of the CTRL, RAD, and BLEO (2 replicates *per* group) were pretreated with 200 U/mL DNase (Worthington) for 30 min at 37°C to remove free‐floating DNA and to digest the DNA of dead cells. The media were then washed out, cells were resuspended in cold PBS for counting, and processed as previously described (Corces et al. 2017). Briefly, a total of 50,000 cells were resuspended in 1 mL of cold ATAC‐seq resuspension buffer (RSB; 10 mM Tris–HCl (pH 7.4), 10 mM NaCl, and 3 mM MgCl_2_ in water). Cells were centrifuged at 500 × *g* for 5 min in a pre‐chilled (4°C) fixed‐angle centrifuge. After centrifugation, 900 μL of supernatant was aspirated, and the remaining 100 μL of supernatant was carefully aspirated by pipetting with a P200 pipette tip to avoid the cell pellet. Cell pellets were then resuspended in 50 μL of ATAC‐seq RSB containing 0.1% NP40, 0.1% Tween‐20, and 0.01% digitonin by pipetting up and down thrice. This cell lysis reaction was incubated on ice for 3 min. After lysis, 1 mL of ATAC‐seq RSB containing 0.1% Tween‐20 (without NP40 or digitonin) was added, and the tubes were inverted to mix. Nuclei were then centrifuged for 10 min at 500 × *g* in a pre‐chilled (4°C) fixed‐angle centrifuge. Supernatant was removed with two pipetting steps, as described before, and nuclei were resuspended in 50 μL of transposition mix (25 μL 2× TD buffer, 2.5 μL transposase (100 nM final), 16.5 μL PBS, 0.5 μL 1% digitonin, 0.5 μL 10% Tween‐20, and 5 μL H_2_O) by pipetting up and down 6 times. The transposition reaction was incubated at 37°C for 30 min. The remainder of the ATAC‐seq library preparation was performed by following the manufacturer's instructions (Nextera DNA Flex Library Prep kit; Illumina, 20,018,704), and the sample quality was validated using the BioAnalyzer2100 (Agilent) before proceeding to deep sequencing. Briefly, all libraries were amplified with a target concentration of 20 μL at 4 nM, which is equivalent to 80 fmol of product. Libraries were purified with the 1.5× AMPure (Beckman) beads and were subjected to next‐generation sequencing. For each condition, duplicate independent experiments were performed.

### 4.6 CUT&RUN and Library Preparation

CUT&RUN was performed following an EpiCypher CUTANA protocol. Concanavalin A beads were activated with cold bead activation buffer. Then, 500,000 cells *per* reaction were collected, followed by two washes in the RT Wash Buffer. Cells were incubated with concanavalin A beads, then resuspended in a cold Antibody (CTCF) Buffer. Then, 1 μg of primary antibody or IgG was added per reaction and incubated overnight at 4°C. Cold digitonin buffer and pAG‐MNase were added to bind to antibody‐tagged chromatin. Subsequently, targeted chromatin was digested by the addition of 100 mM CaCl_2_. Stop Buffer was added to stop MNase enzyme activity. The fragmented chromatin was purified using a CUTANA DNA Purification kit (EpiCypher). For low input cell numbers, the purified DNA was collected by phenol–chloroform, followed by ethanol purification. The final product was quantified with a Qubit fluorometer. For each condition, duplicate independent experiments were performed.

The library preparation used the NEBNext Ultra II DNA Library Prep kit for Illumina (NEB). Less than 30 ng of fragmented DNA was used for library construction. The product was cleaned up with SPRIselect beads (Beckman Coulter). The final products were quantified with Qubit and Agilent Tapestation. Libraries were subsequently sequenced on an Illumina NovaSeq6000.

### 4.7 ATAC‐Seq and CTCF CUT&RUN Data Processing

ATAC‐seq and CTCF CUT&RUN reads were mapped to hg19 with Bowtie2 (version 2.3.5.1). Aligned reads were filtered for a minimum MAPQ of 30, and duplicates were removed using SAMtools (version 1.3.1). The two replicated bam files for each cell line were merged by SAMtools. MACS2 was applied to each merged bam file to call peaks. Then, the bigwig files were generated by deepTools (version 3.5.1) using the RPKM option of the bamCoverage command. The average signals of ATAC‐seq, CTCF, CpG island, repeat elements, and other markers across chromatin higher‐order structure units were obtained using the multiBigwigSummary command from deepTools.^9^

### 4.8 Construction of Hi‐C Libraries

Cells were harvested under 3 biological conditions: CTRL, RAD, and BLEO as indicated above. Hi‐C libraries were prepared using DpnII with minor improvements to increase the final yield. Briefly, 1 million cells per sample were fixed, lysed, and digested overnight with DpnII. First, cell pellets were resuspended in 45 mL of chilled 1× PBS by gently pipetting before 1.25 mL of 37% formaldehyde (1% final concentration) was added. After incubation at room temperature for 15 min, 2.5 mL of 1 M glycine (125 mM final concentration) was added to quench crosslinking. The cell suspension was centrifuged at 2200 × *g* for 10 min at 4°C, and cells were washed with 10 mL of chilled 1× PBS containing 1× EDTA‐free protease inhibitors and PMSF (1 mM final concentration). Coss‐linked cells were centrifuged and resuspended in 1 mL of chilled 1× PBS containing 1× EDTA‐free protease inhibitors and 1 mM PMSF, and transferred to a 1.5 mL Eppendorf tube. Pellet cells were centrifuged at 2200 × *g* for 10 min at 4°C, with the supernatant discarded. In the stage of cellular lysis, 1.25× RE buffer compatible with the restriction enzyme (DpnII) was prepared. During library digestion, 7.5 μL of 20% SDS was added to denature the chromatin before 400 U of the restriction enzyme was used. After fill‐in with biotin‐dCTP (deoxycytidine 5′‐triphosphate), the reactions were ligated, de‐crosslinked, and purified by phenol/chloroform extraction. Biotin was removed from unligated ends, and the DNA was sheared into 100–300 bp fragments using Covaris S2 instrument. Sonicated DNA was ethanol‐precipitated in the presence of glycogen, resuspended, and end‐repaired using the End‐It DNA End‐Repair Kit (Epicentre). The resulting DNA was purified using Agencourt AMPure XP paramagnetic beads (Beckman Coulter) and eluted in molecular grade water. Deoxyadenosine triphosphate (dATP) was added to the DNA ends and subjected to another DNA purification, then biotin‐labeled DNA was pulled down with streptavidin beads (Dynabeads MyOne Streptavidin C1). Pre‐annealed sequencing adapters were ligated to each sample. PCR amplification was performed using Phusion High‐Fidelity DNA Polymerase (New England Biolabs). A final purification step with AMPure XP beads yielded libraries (in the 200–500 bp range), which were then sequenced with a NovaSeq X Plus instrument. Products were digested with NheI and DpnII to quantify the frequency of introducing new chimeric restriction sites in the fill‐in reactions, a step used as quality control. Aliquots of the final libraries were cloned using the Zero Blunt TOPO PCR Cloning Kit for sequencing and Sanger‐sequenced (50 clones *per* library) to evaluate the library content. For each condition, duplicate independent experiments were performed.

### 4.9 Hi‐C Sequence Data Processing

The raw paired‐end sequencing reads were aligned to the hg19 reference genome, processed, and ICE normalized using the HiC‐Pro (version 3.1.0) pipeline. The raw contact matrices were generated for each replicate at binning resolutions of 5 kb, 10 kb, 20 kb, 40 kb, 100 kb, and 500 kb. To correct bias, the raw contact matrices were normalized using the iterative correction and eigenvector decomposition (ICE) method. To generate the maximum counts of reads and ensure comparability between samples, we pooled two biological replicates for each cell line after data quality and reproducibility checks, and valid pairs were down‐sampled equally to 340 million for subsequent analyses. For obtaining the interaction data stored in different formats (hic, h5, and cool), we used the hicConvertFormat command from HiCExplorer (version 3.7.2) to convert the output files generated by HiC‐Pro into the desired formats. As for relative contact probability analysis, the raw contact sparse matrix with 100 kb resolution was applied to calculate the contact probability (*p(s)*) relative to the genomic distance. We counted the interactions in each bin and calculated *p(s)* by dividing the number of interactions in each bin by the total number of interactions across all bins. For the analysis of OIS and RS Hi‐C data, we employed the same reference genome and processing pipeline as used in this study. Visualization of regions of interest was performed by generating heatmaps of Hi‐C interaction matrices using the pheatmap package in R and the TreeView tool (http://jtreeview.sourceforge.net/).

### 4.10 Chromatin A/B Compartments and Compartment Strength

The principal component analysis (PCA) was performed by ICE Hi‐C matrices at a resolution of 100 kb using cooltools and stratified by GC content (Abdennur et al. 2024). The first principal component (PC1) values were used to define A/B compartments, with the positive values representing A compartment regions and negative values representing B compartment regions. Saddle plots were generated by GENOVA to assign each bin to its corresponding percentile value and divide the genome into fifty sets of deciles. Measuring the compartmentalization strength was to compare the average observed/expected values between inter‐ and intra‐compartment. The distance‐dependent chromatin interactions and compartmentalization were quantified by Pentad, which could be used to quantify and visualize compartment strength by extracting interaction submatrices corresponding to different combinations of compartment types from the averaged observed/expected Hi‐C contact maps, rescaling them to a uniform size, and quantifying inter‐ and intra‐compartment strength for comparative analysis.

### 4.11 TAD Analysis

Insulation score was calculated by matrix2insulation.pl public script using ICE normalized Hi‐C matrices at 40 kb resolution. TADs were identified using the hicFindTADs command in HiCExplorer. TADs can be investigated globally by aggregating Hi‐C matrix around TADs. Using coolpuppy (version 1.1.0) to perform aggregate TAD analysis (ATA) without the first two diagonals and visualize the heatmaps. The consolidation score quantifies the consolidation levels of TADs, which is defined as the ratio of average interaction frequency within each TAD (excluding short‐distance interactions less than 400 kb) and the local background interaction frequency from nearby non‐TAD regions. To assess the organizational state of important chromatin interactions within TAD, we use 10 kb resolution matrices to calculate the TAD degree of disorder (DoD) values by MDkNN (version 0.0.1). The higher the DoD value, the more unstable the TAD was. The TAD connectivity score was defined as the ratio of the total number of PETs within a TAD to the total number of PETs with one end located within that TAD. Differential TADs were identified using cLoops2 (version 0.0.3), with TADs from non‐senescent cells serving as the reference. Briefly, the method involves two‐pass Mahalanobis distance calculation followed by chi‐square testing to detect significantly altered TADs; those with *p* value less than 0.05 were considered as differential TADs. TAD rearrangement events were examined using bedtools (version 2.31.0). To assign TADs to specific compartments, we performed positional analysis between TADs and compartments using bedtools. If a TAD spanned different types of compartments, it was assigned proportionally based on the relative lengths of the A and B compartments it overlapped.

### 4.12 Chromatin Loop Calling and Analysis

We detected loops using HiCCUPS at 5 kb, 10 kb, and 25 kb resolutions with fdr 0.1 for each sample. Identification of differential loops was performed using cLoops2. First, loops from HiCCUPS were quantified using the quant module, followed by differential analysis using the callDiffLoops command. Stringent parameters were applied for differential loop detection: ‐‐customize ‐cacut 5 ‐cmcut 0.5. We performed aggregated peak analysis (APA) using coolpuppy at ICE, normalized to 10 kb resolution and 100 kb window, without the first two diagonals. Values of enrichment of pileups were the enrichment of interactions in the center pixel of the matrix. The spatial relationships between loops or differential loops and compartments, TADs, and CTCF binding sites were determined using bedtools.

### 4.13 Detection of Differential Chromatin Interactions Within TAD Rearrangement Events and 3D Modeling for SASP Factor Location

To investigate chromatin rewiring during TIS, we first identified differential chromatin interactions (DCIs) at 10 kb resolution using multiHiCcompare (version 1.20.0), a cyclic loess regression‐based joint normalization technique for removing biases across non‐senescent and senescent Hi‐C datasets in this study. The fastlo function was used to normalize the Hi‐C matrices, and hic_exactTest was used to determine significant DCIs using the fdr argument with log_2_CPM > 2, absolute log_2_(fold change) > 1.2, and adjusted *p* value < 0.05. To investigate the role of chromatin interactions in local spatial remodeling, we focused on differential DCIs that were shorter than 2 Mb within the defined TAD rearrangement events. For each TAD and its flanking 80 kb regions, we quantified the number of upregulated and downregulated differential DCIs, retaining only TADs with at least 10 up‐ or downregulated DCIs for downstream analysis. We then calculated the proportion of up‐ and downregulated DCIs within each TAD, along with the average signal intensities of PC1, ATAC‐seq, CTCF, repeat elements, and other relevant markers. Finally, Spearman correlation coefficients were computed between the percentage of down‐regulated DCIs and each of these indicators. To evaluate the relationship between downregulated DCIs and chromatin compartments, TADs were classified into high (≥ 0.5) or low (< 0.5) groups based on the fraction of downregulated. For each sample, the association between compartment identity based on PC1 values and the downregulated DCI group was assessed using odds ratios and Fisher's exact test. A logistic regression model was used to assess the association between TAD rearrangement types and the likelihood of high downDCI group, with unchanged as the reference category. Separate models were fitted for RAD and BLEO samples, and odds ratios were computed from model coefficients. We used ParticleChromo3D (Vadnais et al. 2022), a particle swarm optimization algorithm, to predict the 3D chromatin structure of TADs containing SASP factors defined by RNA‐seq, along with their adjacent TADs. The resulting models were visualized using the Rpdb package in R.

### 4.14 Statistics and Reproducibility

Standard statistical analyses were performed using R. The type of statistical tests to use and the test results were described in the figure legend. No experimental samples were excluded from statistical analysis. We performed reproducible checks and hierarchical clustering on Hi‐C data using a 100 kb raw matrix restricted in 5 Mb by HiCRep, PC1 values at 100 kb resolution, and insulation score profiles at 40 kb resolution.

## Author Contributions

G.Z., C.W. and Y.S. conceived this study, designed the experiments, and orchestrated the project. W.Z. and Z.J. performed most of the in vitro assays. G.Z. analyzed data and wrote part of the manuscript. Q.X. helped with cell culture and drug treatment. H.L. and G.W. provided technical support and feedback regarding the work. Y.S. supervised the study, provided funding support and conceptual input, and finalized the manuscript. All authors critically read and commented on the final manuscript.

## Funding

This work was supported by the Chinese Academy of Sciences, 82130045, 82350710221, 82571777.

## Conflicts of Interest

The authors declare no conflicts of interest.

## Supporting information


**Data S1:** Supplementary Figures.

**Data S2:** Supplementary Tables.

## Data Availability

Hi‐C data in proliferating and senescent PSC27 cells were deposited in the Gene Expression Omnibus (GEO) database under accession code GSE193429. ATAC‐seq data were deposited in GEO under the code GSE277466. RNA‐seq data referred to the former datasets in GSE128282 and GSE190278. CTCF‐based CUT&Tag data were deposited in GEO under the code GSE252521. H3K9me3 ChIP‐seq data referred to the former datasets in GSE163105. Requests for further information and reagents should be directed to and will be fulfilled by the corresponding author. Primary cell line and associated reagents generated in this study are available upon reasonable request, and with a completed Materials Transfer Agreement if there is a potential for commercial application.
