# Correction to “Spatial Reorganization of Chromatin Architecture Shapes the Expression Phenotype of Therapy‐Induced Senescent Cells”

**DOI:** 10.1111/acel.70427

**Published:** 2026-02-23

Zhang, G., W. Zhang, C. Wang, et al. 2026. “Spatial Reorganization of Chromatin Architecture Shapes the Expression Phenotype of Therapy‐Induced Senescent Cells.” *Aging Cell* 25, no. 1: e70366. https://doi.org/10.1111/acel.70366.

In the published version of the above article, we identified several minor errors, including small wording and formatting issues. These have now been corrected as detailed below. This correction does not affect the results or conclusions of the article.

**Main text:**

(1) In section 2.3, third paragraph, in the following sentence:

‘Across short‐and long‐range interactions within both A and B compartments, RAD showed significantly weaker compartmentalization than CTRL, whereas BLEO exhibited significantly stronger compartmentalization in short‐range A and long‐range B compared with CTRL (Figure 3b and Table S3).’ **“and BLEO”** should be added after **“RAD”** and the term **“CTRL”** should be corrected to **“RAD”**.

The corrected sentence should now read as follows:

Across short‐and long‐range interactions within both A and B compartments, RAD **and BLEO** showed significantly weaker compartmentalization than CTRL, whereas BLEO exhibited significantly stronger compartmentalization in short‐range A and long‐range B compared with **RAD** (Figure 3b and Table S3).

(2) In section 2.5 third paragraph, in the following sentence:

‘Approximately 66% of loops in all 3 conditions fell into the AA type, which was also the predominant configuration across different loop lengths (Figures 5d and S7b)’.

**‘(Figures 5d and S7b)’** should be corrected to **‘(Figures 5e and S7b)’**.

(3) In section 2.8, page 12, third paragraph, the phrase **“and Figure S11d”** should be added after **“Figure 6d”**.

(4) In the Methods section, subsection 4.9, first paragraph, in the following sentence,

‘For the analysis of OIS and RS Hi‐C data, we employed the same reference genome and processing pipeline as used in this study.’ the phrase **“OIS and RS”** should be corrected to **“OIS, RS and deep RS”** and the corrected sentence should now read as

**‘**For the analysis of OIS, RS and deep RS Hi‐C data, we employed the same reference genome and processing pipeline as used in this study’.

(5) In section 4.12, line 2, the term **“fdr 0.1”** should be corrected to **“FDR < 0.1”**.

(6) In the Data Availability Statement, the term **“CUT&Tag”** should be corrected to **“CUT&RUN”** and the sentence **“H3K9me3 ChIP‐seq data referred to former datasets in GSE163105.”** should be removed, as this dataset was not used in the study.

(7) In Figure caption 2, lines 1–2, the chromosome number in **Figure 2a and b** should be **“4”** instead of **“11”** and in **Figure 2i**, the phrase **“change of intra‐ and inter‐ interactions”** should be corrected to **“changes of interactions.”**

(8) In Figure **5a**, line 2, the term “The top **right** corner” should be corrected to “The top **left** corner”.

(9) In Figure **6a**, the term **“reorganization”** should be corrected to **“rearrangement events”**

(10) In **Figure 6f**, the sentence **“Euclidean distance between the location of NRG1 and other locations in the TAD represented by the blue line segment.”** should be corrected to **“Boxplot of comparison of Euclidean distance between the location of NRG1 in red and other locations represented by the blue line segment in e.”**

(11) **Figure 5e** appeared to be distorted, and a legend title was inadvertently added to **Figure 5i**. The corrected version of Figure 5 was shown below

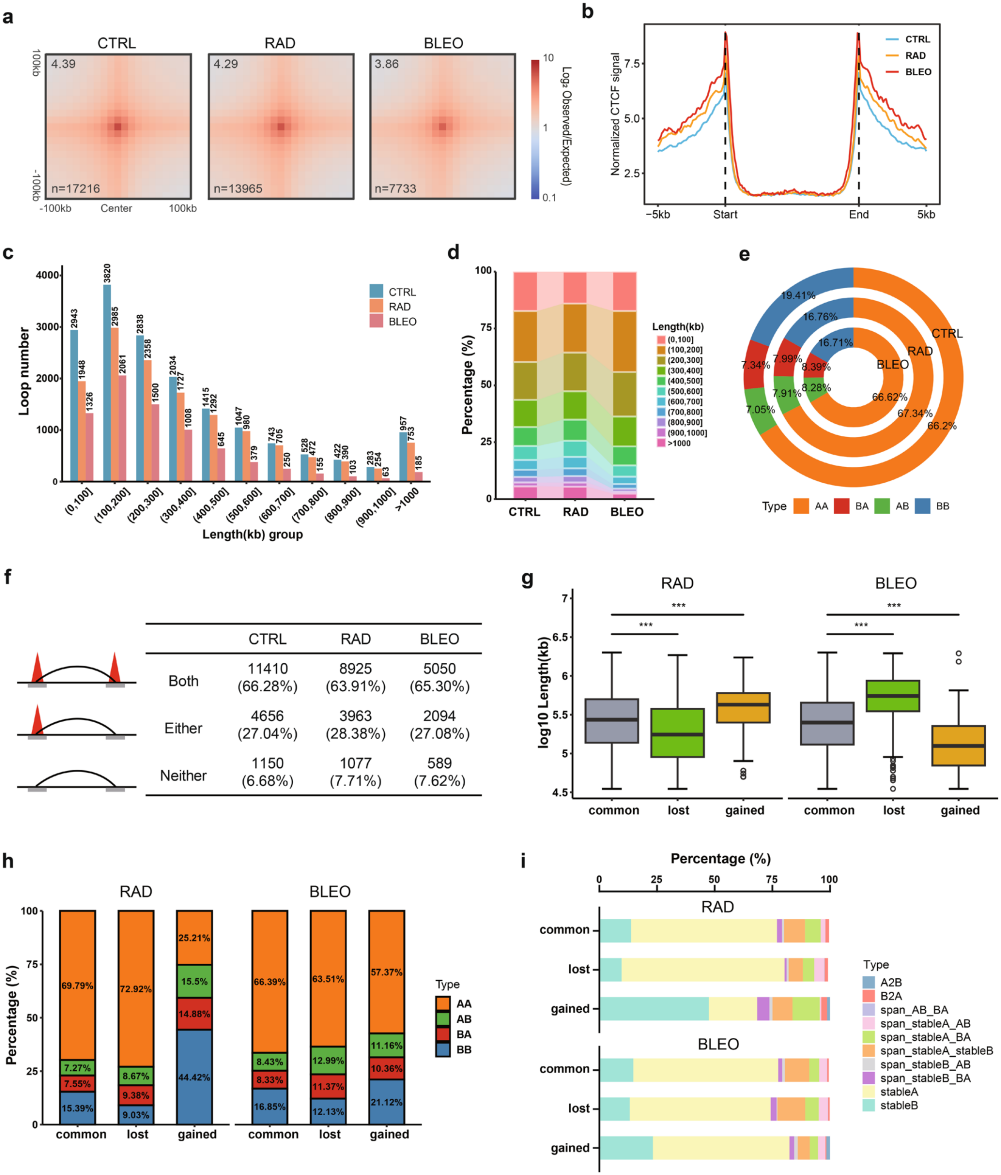

(12) The incorrect version of **Figure 7** was updated in the published version, the trend shown in the figure did not match the results described in the article. The corrected version of **Figure 7** was shown below

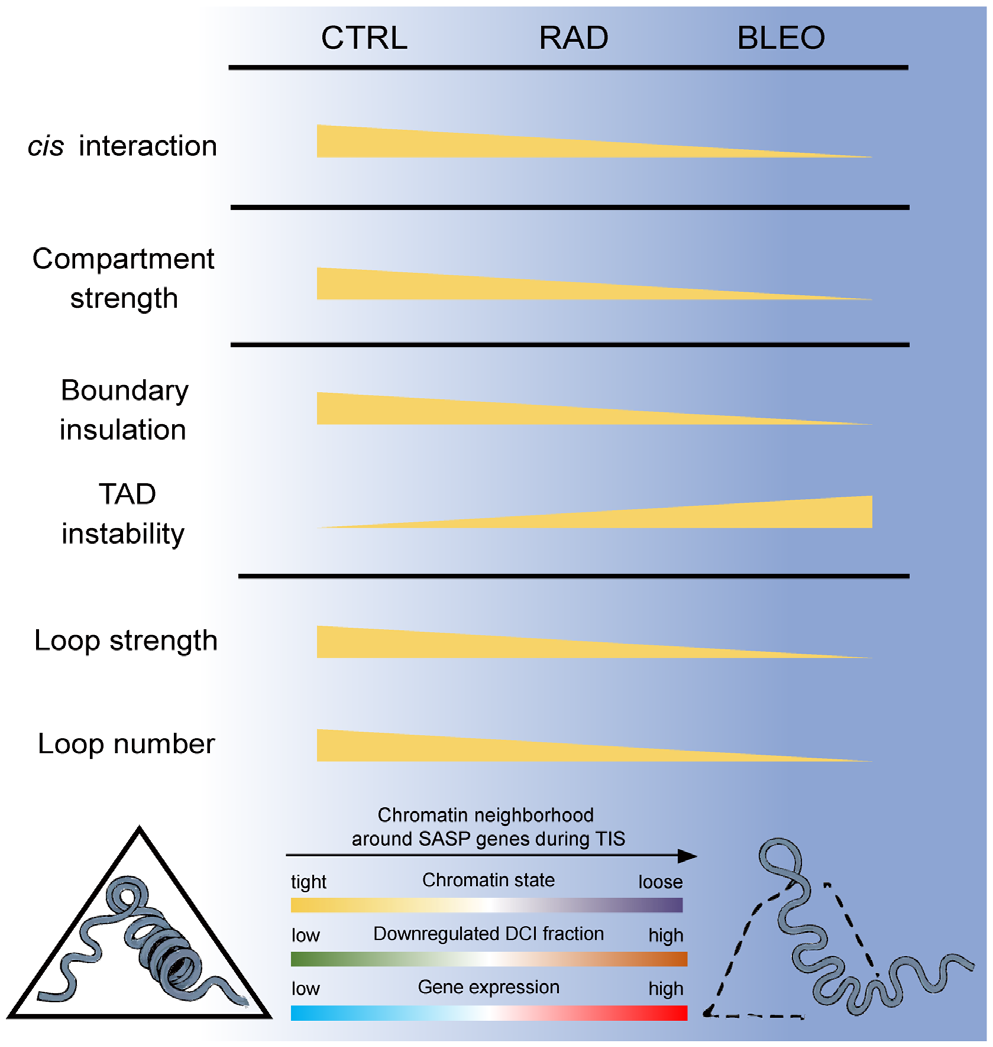

(13) We missed to include the reference citations to bioinformatics tools and the references in reference list. The citations and the missed out references were listed below:

- In **Methods section, subsection 4.3**, in second paragraph, (Kim et al. 2015) should be cited after Hisat2 (version 2.2.1), (Anders et al. 2015) after HTSeq (version 2.0.2), (Pertea et al. 2015) after StringTie (version 2.2.1), (Love et al. 2014) after DESeq2 (version 1.34.0) and (Dennis Jr. et al. 2003) after DAVID.
- **In subsection 4.7**, (Langmead and Salzberg 2012) should be cited after Bowtie2 (version 2.3.5.1), (Li et al. 2009) after SAMtools (version 1.3.1), (Feng et al. 2012) should be cited at the end of the text ‘MACS2 was applied to each merged bam file to call peaks’, (Ramírez et al. 2014) after deepTools (version 3.5.1).
- In subsection 4.9, (Servant et al. 2015) should be cited after HiC‐Pro (version 3.1.0) pipeline, (Ramírez et al. 2018) after HiCExplorer (version 3.7.2).
- In subsection 4.10, (van der Weide et al. 2021) should be cited after ‘GENOVA’, (Magnitov et al. 2022) after ‘Pentad’.
- In subsection 4.11, (Flyamer et al. 2020) should be cited after ‘coolpuppy (version 1.1.0)’, (Cao et al. 2022) after ‘cLoops2 (version 0.0.3)’.
- In subsection 4.13, (Stansfield et al. 2019) **should be cited after ‘multiHiCcompare (version 1.20.0)’**.
- **In subsection 4.14, (**Yang et al. 2017 **) should be cited after ‘**HiCRep’.

We apologize for these errors.

## References

[acel70427-bib-0001] Anders, S. , P. T. Pyl , and W. Huber . 2015. “HTSeq—A Python Framework to Work With High‐Throughput Sequencing Data.” Bioinformatics (Oxford, England) 31: 166–169. 10.1093/bioinformatics/btu638. 25260700 PMC4287950

[acel70427-bib-0002] Cao, Y. , S. Liu , G. Ren , Q. Tang , and K. Zhao . 2022. “cLoops2: A Full‐Stack Comprehensive Analytical Tool for Chromatin Interactions.” Nucleic Acids Research 50: 57–71. 10.1093/nar/gkab1233. 34928392 PMC8754654

[acel70427-bib-0003] Dennis, G., Jr. , B. T. Sherman , D. A. Hosack , et al. 2003. “DAVID: Database for Annotation, Visualization, and Integrated Discovery.” Genome Biology 4: P3.12734009

[acel70427-bib-0004] Feng, J. , T. Liu , B. Qin , Y. Zhang , and X. S. Liu . 2012. “Identifying ChIP‐seq Enrichment Using MACS.” Nature Protocols 7: 1728–1740. 10.1038/nprot.2012.101. 22936215 PMC3868217

[acel70427-bib-0005] Flyamer, I. M. , R. S. Illingworth , and W. A. Bickmore . 2020. “Coolpup.py: Versatile Pile‐Up Analysis of Hi‐C Data.” Bioinformatics (Oxford, England) 36: 2980–2985. 10.1093/bioinformatics/btaa073. 32003791 PMC7214034

[acel70427-bib-0006] Kim, D. , B. Langmead , and S. L. Salzberg . 2015. “HISAT: A Fast Spliced Aligner With Low Memory Requirements.” Nature Methods 12: 357–360. 10.1038/nmeth.3317. 25751142 PMC4655817

[acel70427-bib-0007] Langmead, B. , and S. L. Salzberg . 2012. “Fast Gapped‐Read Alignment with Bowtie 2.” Nature Methods 9: 357–359. 10.1038/nmeth.1923. 22388286 PMC3322381

[acel70427-bib-0008] Li, H. , B. Handsaker , A. Wysoker , et al. 2009. “The Sequence Alignment/Map Format and SAMtools.” Bioinformatics (Oxford, England) 25: 2078–2079. 10.1093/bioinformatics/btp352. 19505943 PMC2723002

[acel70427-bib-0009] Love, M. I. , W. Huber , and S. Anders . 2014. “Moderated Estimation of Fold Change and Dispersion for RNA‐Seq Data With DESeq2.” Genome Biology 15: 550. 10.1186/s13059-014-0550-8. 25516281 PMC4302049

[acel70427-bib-0010] Magnitov, M. D. , A. K. Garaev , A. V. Tyakht , S. V. Ulianov , and S. V. Razin . 2022. “Pentad: A Tool for Distance‐Dependent Analysis of Hi‐C Interactions Within and Between Chromatin Compartments.” BMC Bioinformatics 23: 116. 10.1186/s12859-022-04654-6. 35366792 PMC8976968

[acel70427-bib-0011] Pertea, M. , G. M. Pertea , C. M. Antonescu , T. C. Chang , J. T. Mendell , and S. L. Salzberg . 2015. “StringTie Enables Improved Reconstruction of a Transcriptome From RNA‐Seq Reads.” Nature Biotechnology 33: 290–295. 10.1038/nbt.3122. PMC4643835 25690850

[acel70427-bib-0012] Ramírez, F. , V. Bhardwaj , L. Arrigoni , et al. 2018. “High‐Resolution TADs Reveal DNA Sequences Underlying Genome Organization in Flies.” Nature Communications 9: 189. 10.1038/s41467-017-02525-w. PMC5768762 29335486

[acel70427-bib-0013] Ramírez, F. , F. Dündar , S. Diehl , B. A. Grüning , and T. Manke . 2014. “deepTools: A Flexible Platform for Exploring Deep‐Sequencing Data.” Nucleic Acids Research 42: W187–W191. 10.1093/nar/gku365. 24799436 PMC4086134

[acel70427-bib-0014] Servant, N. , N. Varoquaux , B. R. Lajoie , et al. 2015. “HiC‐Pro: An Optimized and Flexible Pipeline for Hi‐C Data Processing.” Genome Biology 16: 259. 10.1186/s13059-015-0831-x. 26619908 PMC4665391

[acel70427-bib-0015] Stansfield, J. C. , K. G. Cresswell , and M. G. Dozmorov . 2019. “multiHiCcompare: Joint Normalization and Comparative Analysis of Complex Hi‐C Experiments.” Bioinformatics (Oxford, England) 35: 2916–2923. 10.1093/bioinformatics/btz048. 30668639 PMC6736119

[acel70427-bib-0016] van der Weide, R. H. , T. van den Brand , J. H. I. Haarhuis , H. Teunissen , B. D. Rowland , and E. de Wit . 2021. “Hi‐C Analyses With GENOVA: A Case Study With Cohesin Variants.” NAR Genomics and Bioinformatics 3: lqab040. 10.1093/nargab/lqab040. 34046591 PMC8140737

[acel70427-bib-0017] Yang, T. , F. Zhang , G. G. Yardımcı , et al. 2017. “HiCRep: Assessing the Reproducibility of Hi‐C Data Using a Stratum‐Adjusted Correlation Coefficient.” Genome Research 27: 1939–1949. 10.1101/gr.220640.117. 28855260 PMC5668950

