# Supplementary material for: Spatial Reorganization of Chromatin Architecture Shapes the Expression Phenotype of Therapy‐Induced Senescent Cells
Source: Aging Cell. 2026 Jan 6;25(1):e70366. doi: 10.1111/acel.70366 (PMC12771664; doi:10.1111/acel.70366)
Supplement: Supplementary file 1 — Data S1: Supplementary Figures. [file ACEL-25-e70366-s002.pptx]

## Slide 1
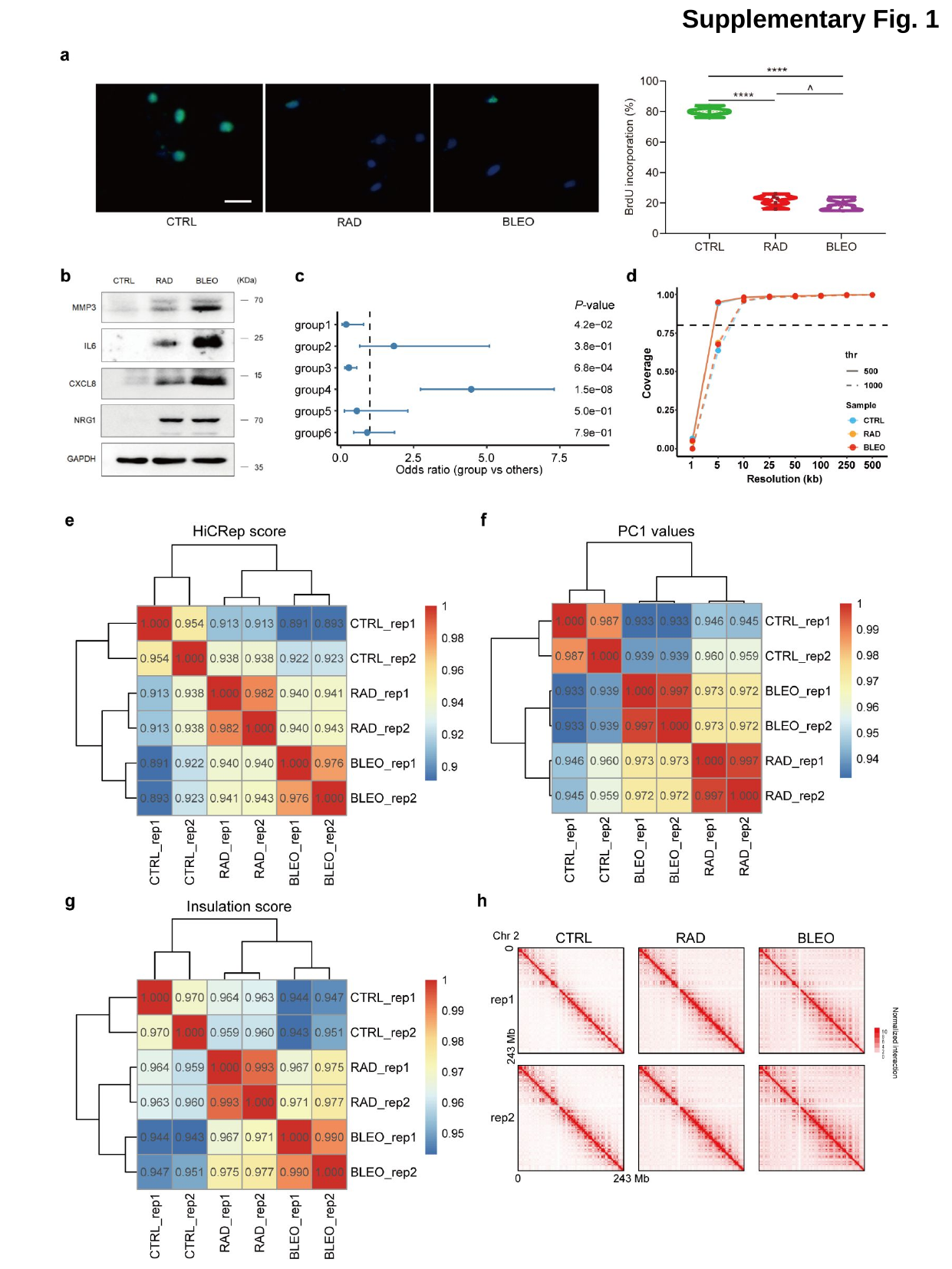

Supplementary Fig. 1

## Slide 2
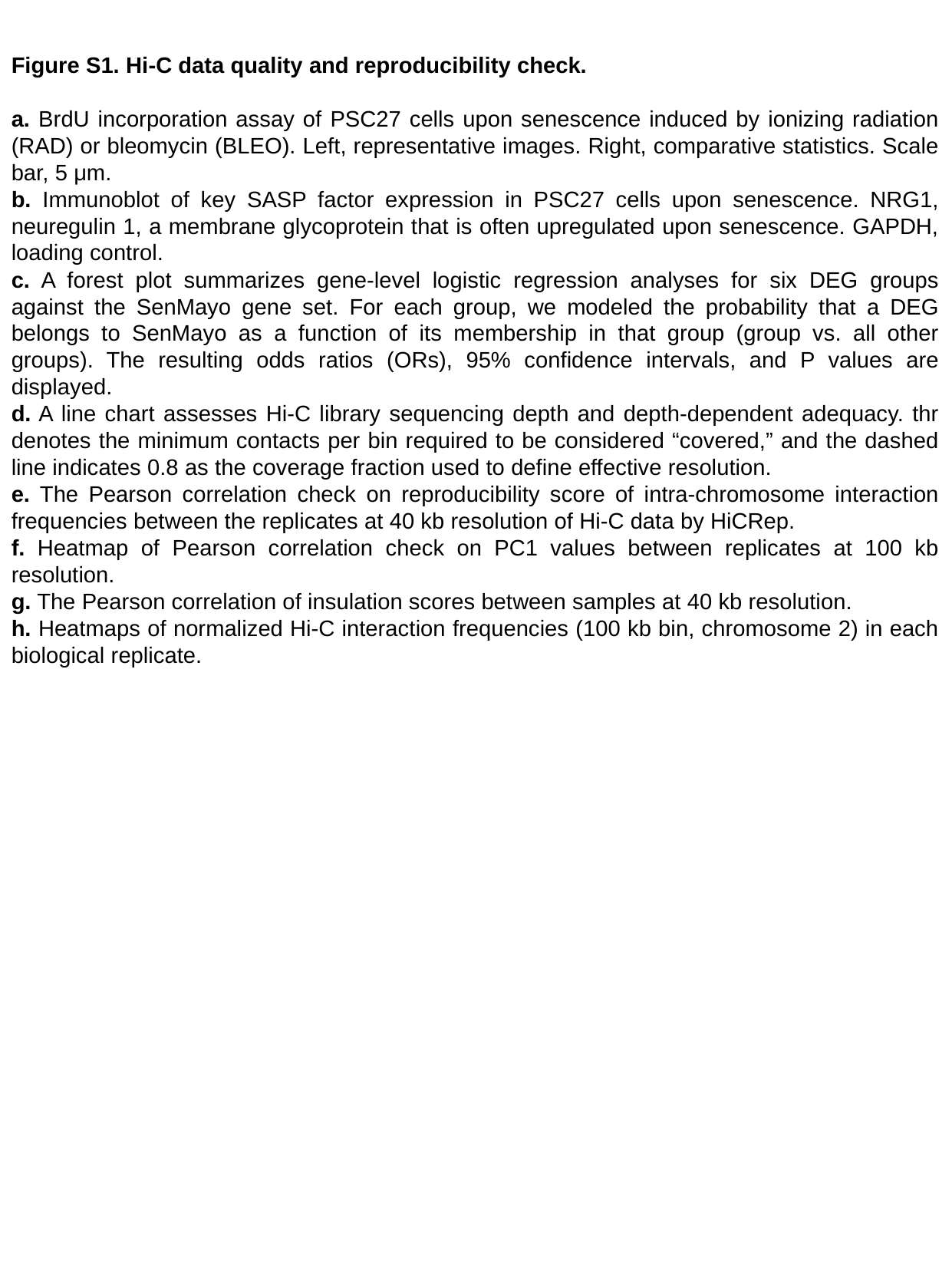

Figure S1. Hi-C data quality and reproducibility check.
a. BrdU incorporation assay of PSC27 cells upon senescence induced by ionizing radiation (RAD) or bleomycin (BLEO). Left, representative images. Right, comparative statistics. Scale bar, 5 μm.
b. Immunoblot of key SASP factor expression in PSC27 cells upon senescence. NRG1, neuregulin 1, a membrane glycoprotein that is often upregulated upon senescence. GAPDH, loading control.
c. A forest plot summarizes gene-level logistic regression analyses for six DEG groups against the SenMayo gene set. For each group, we modeled the probability that a DEG belongs to SenMayo as a function of its membership in that group (group vs. all other groups). The resulting odds ratios (ORs), 95% confidence intervals, and P values are displayed.
d. A line chart assesses Hi-C library sequencing depth and depth-dependent adequacy. thr denotes the minimum contacts per bin required to be considered “covered,” and the dashed line indicates 0.8 as the coverage fraction used to define effective resolution.
e. The Pearson correlation check on reproducibility score of intra-chromosome interaction frequencies between the replicates at 40 kb resolution of Hi-C data by HiCRep.
f. Heatmap of Pearson correlation check on PC1 values between replicates at 100 kb resolution.
g. The Pearson correlation of insulation scores between samples at 40 kb resolution.
h. Heatmaps of normalized Hi-C interaction frequencies (100 kb bin, chromosome 2) in each biological replicate.

## Slide 3
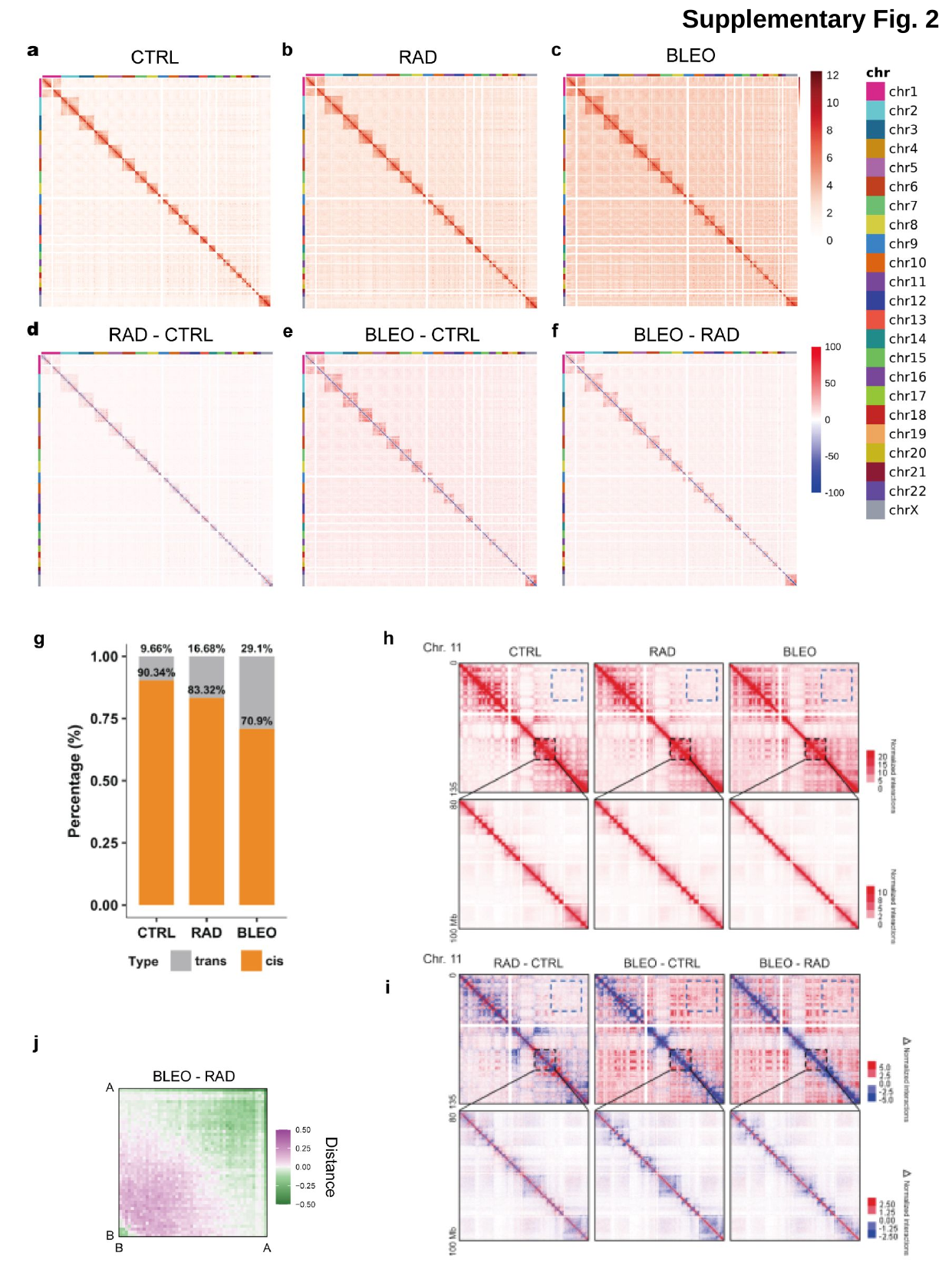

Supplementary Fig. 2

## Slide 4
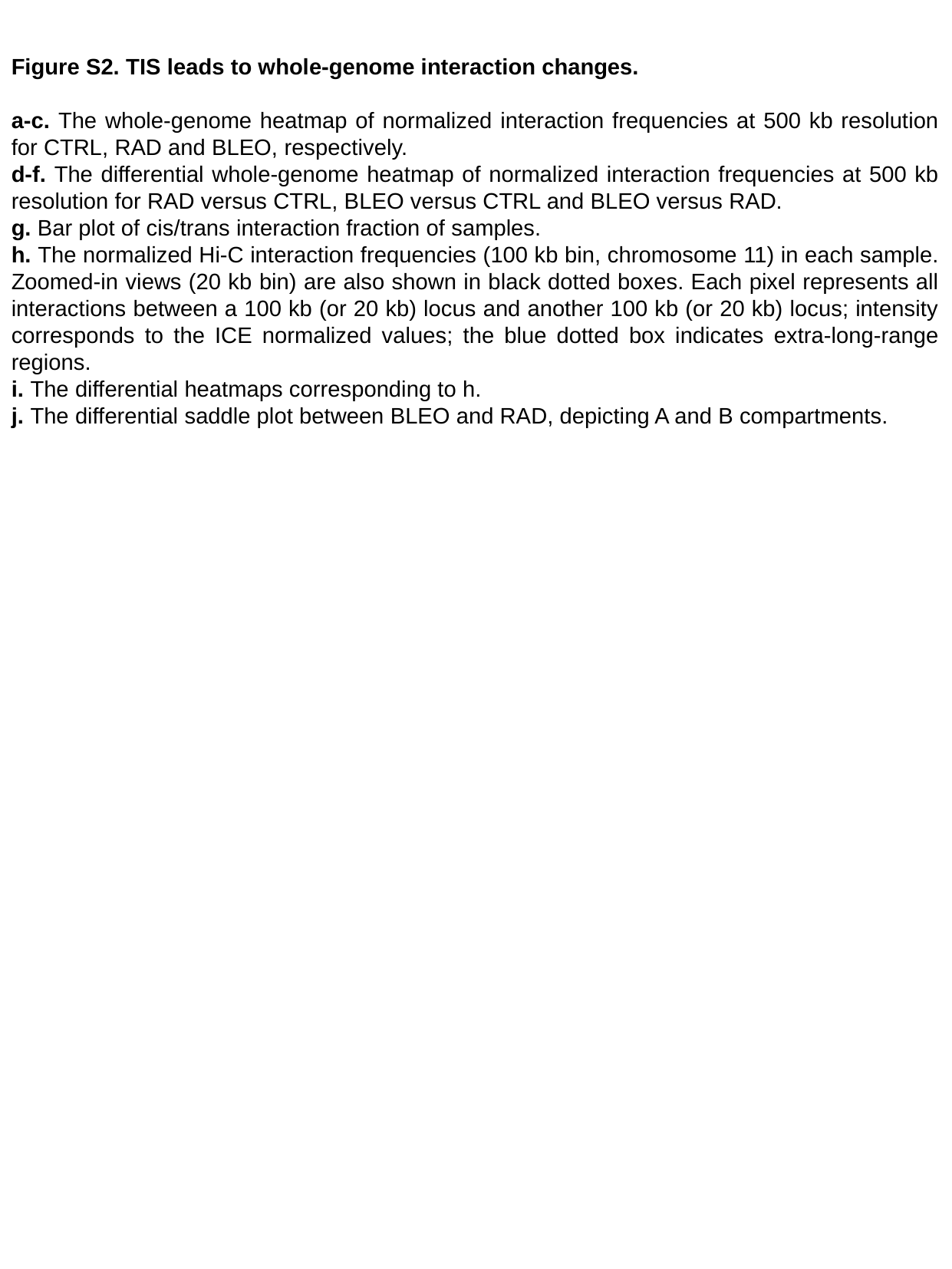

Figure S2. TIS leads to whole-genome interaction changes.
a-c. The whole-genome heatmap of normalized interaction frequencies at 500 kb resolution for CTRL, RAD and BLEO, respectively.
d-f. The differential whole-genome heatmap of normalized interaction frequencies at 500 kb resolution for RAD versus CTRL, BLEO versus CTRL and BLEO versus RAD.
g. Bar plot of cis/trans interaction fraction of samples.
h. The normalized Hi-C interaction frequencies (100 kb bin, chromosome 11) in each sample. Zoomed-in views (20 kb bin) are also shown in black dotted boxes. Each pixel represents all interactions between a 100 kb (or 20 kb) locus and another 100 kb (or 20 kb) locus; intensity corresponds to the ICE normalized values; the blue dotted box indicates extra-long-range regions.
i. The differential heatmaps corresponding to h.
j. The differential saddle plot between BLEO and RAD, depicting A and B compartments.

## Slide 5
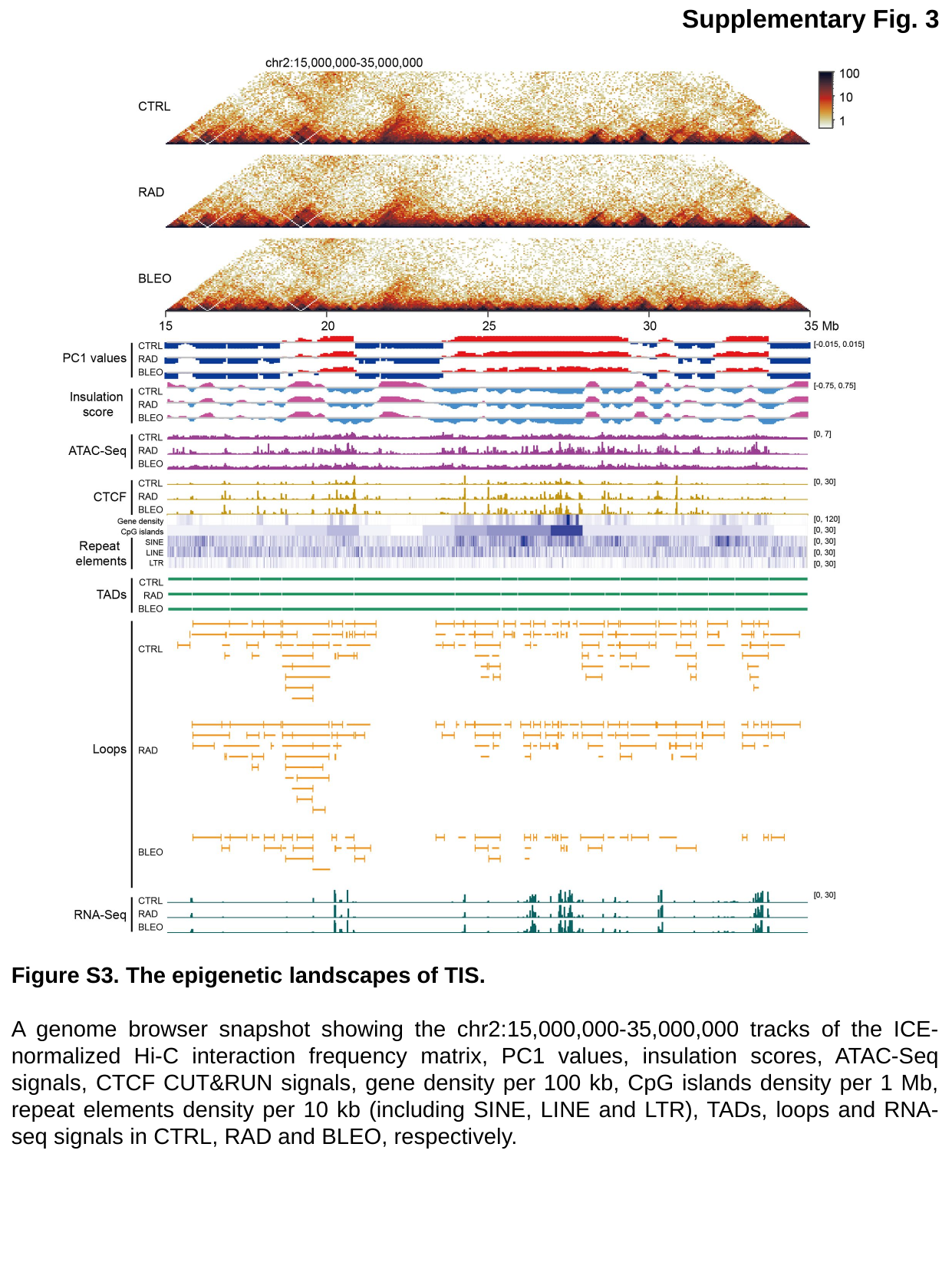

Supplementary Fig. 3
Figure S3. The epigenetic landscapes of TIS.
A genome browser snapshot showing the chr2:15,000,000-35,000,000 tracks of the ICE-normalized Hi-C interaction frequency matrix, PC1 values, insulation scores, ATAC-Seq signals, CTCF CUT&RUN signals, gene density per 100 kb, CpG islands density per 1 Mb, repeat elements density per 10 kb (including SINE, LINE and LTR), TADs, loops and RNA-seq signals in CTRL, RAD and BLEO, respectively.

## Slide 6
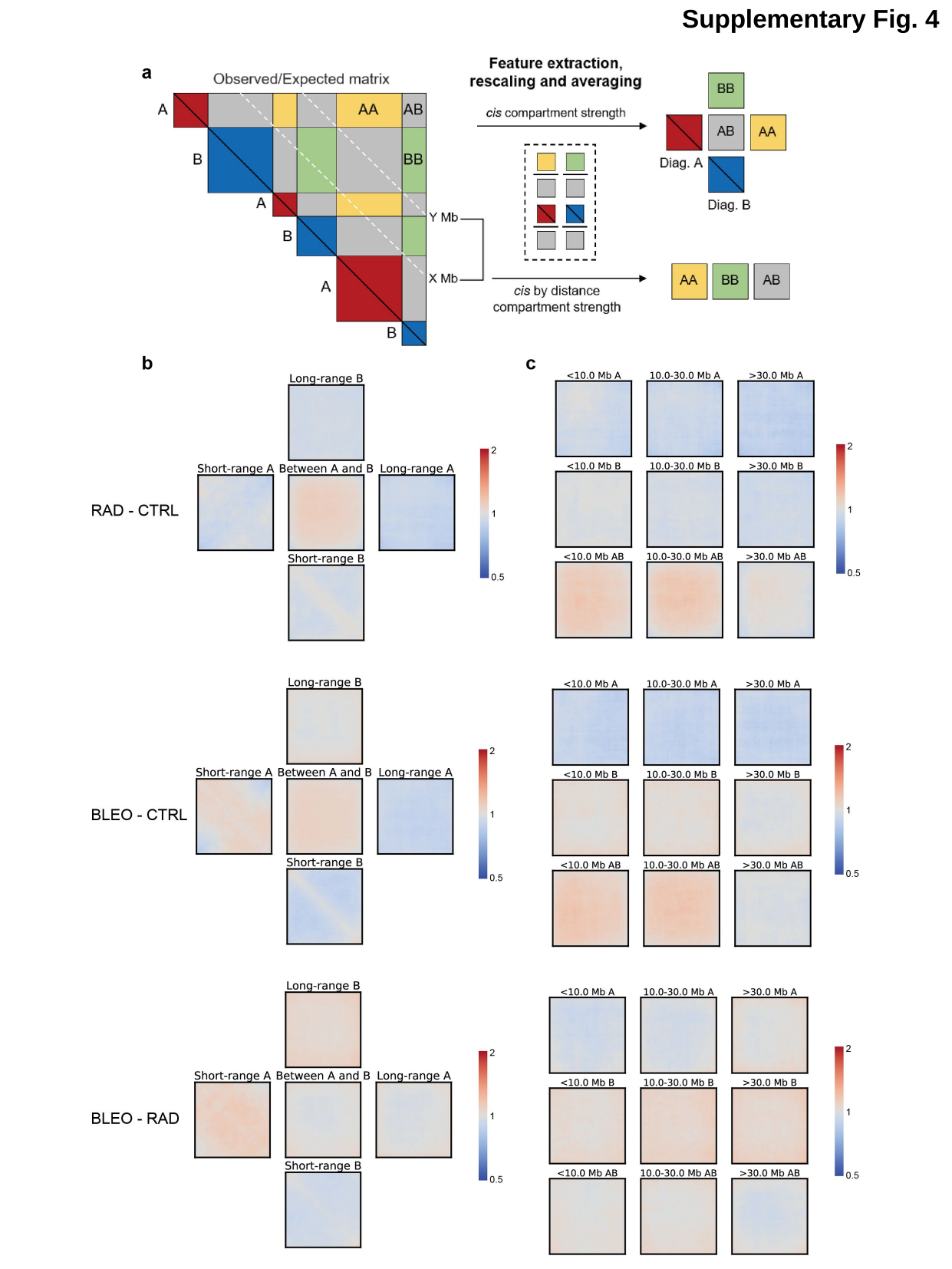

Supplementary Fig. 4

## Slide 7
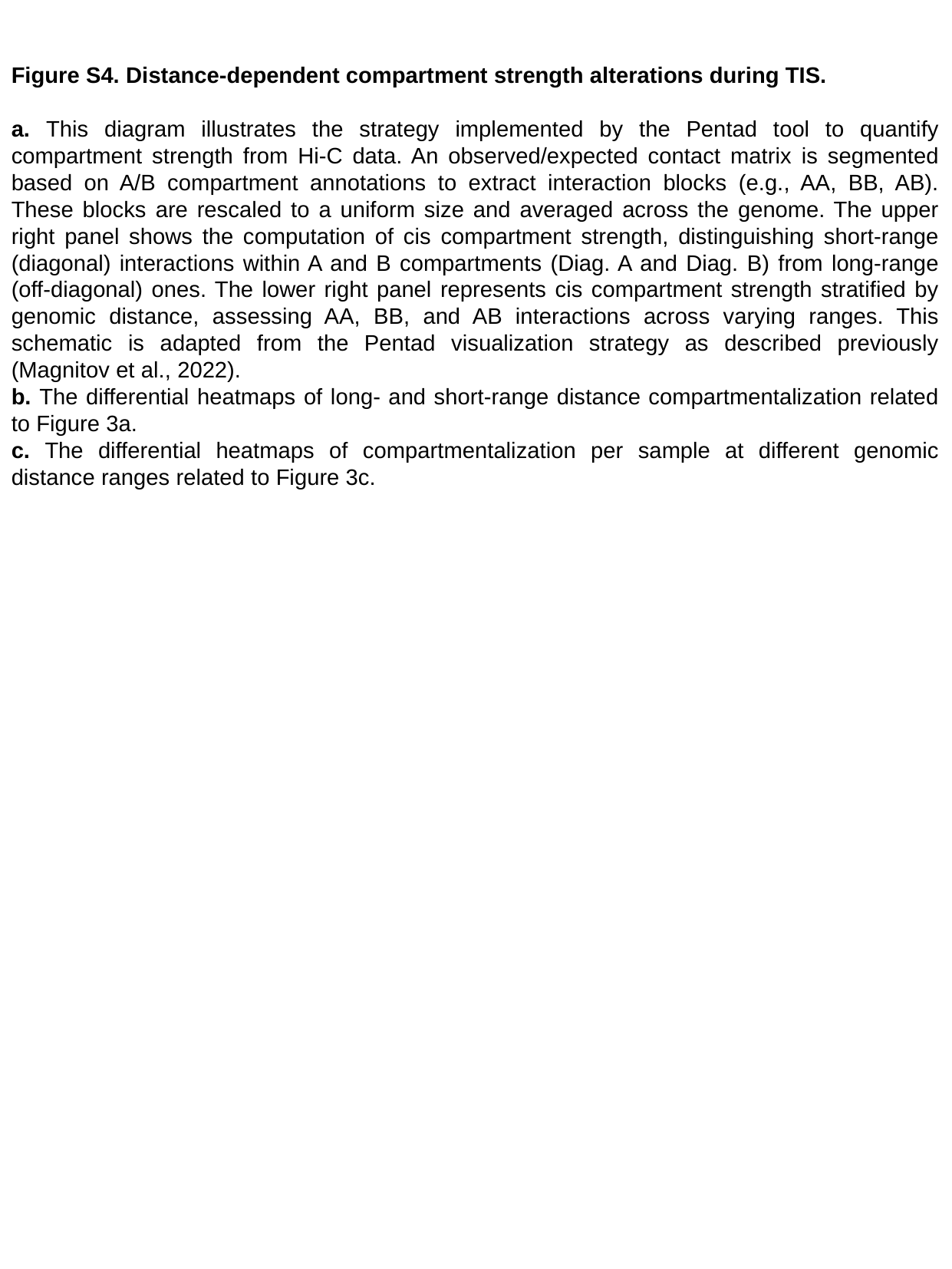

Figure S4. Distance-dependent compartment strength alterations during TIS.
a. This diagram illustrates the strategy implemented by the Pentad tool to quantify compartment strength from Hi-C data. An observed/expected contact matrix is segmented based on A/B compartment annotations to extract interaction blocks (e.g., AA, BB, AB). These blocks are rescaled to a uniform size and averaged across the genome. The upper right panel shows the computation of cis compartment strength, distinguishing short-range (diagonal) interactions within A and B compartments (Diag. A and Diag. B) from long-range (off-diagonal) ones. The lower right panel represents cis compartment strength stratified by genomic distance, assessing AA, BB, and AB interactions across varying ranges. This schematic is adapted from the Pentad visualization strategy as described previously (Magnitov et al., 2022).
b. The differential heatmaps of long- and short-range distance compartmentalization related to Figure 3a.
c. The differential heatmaps of compartmentalization per sample at different genomic distance ranges related to Figure 3c.

## Slide 8
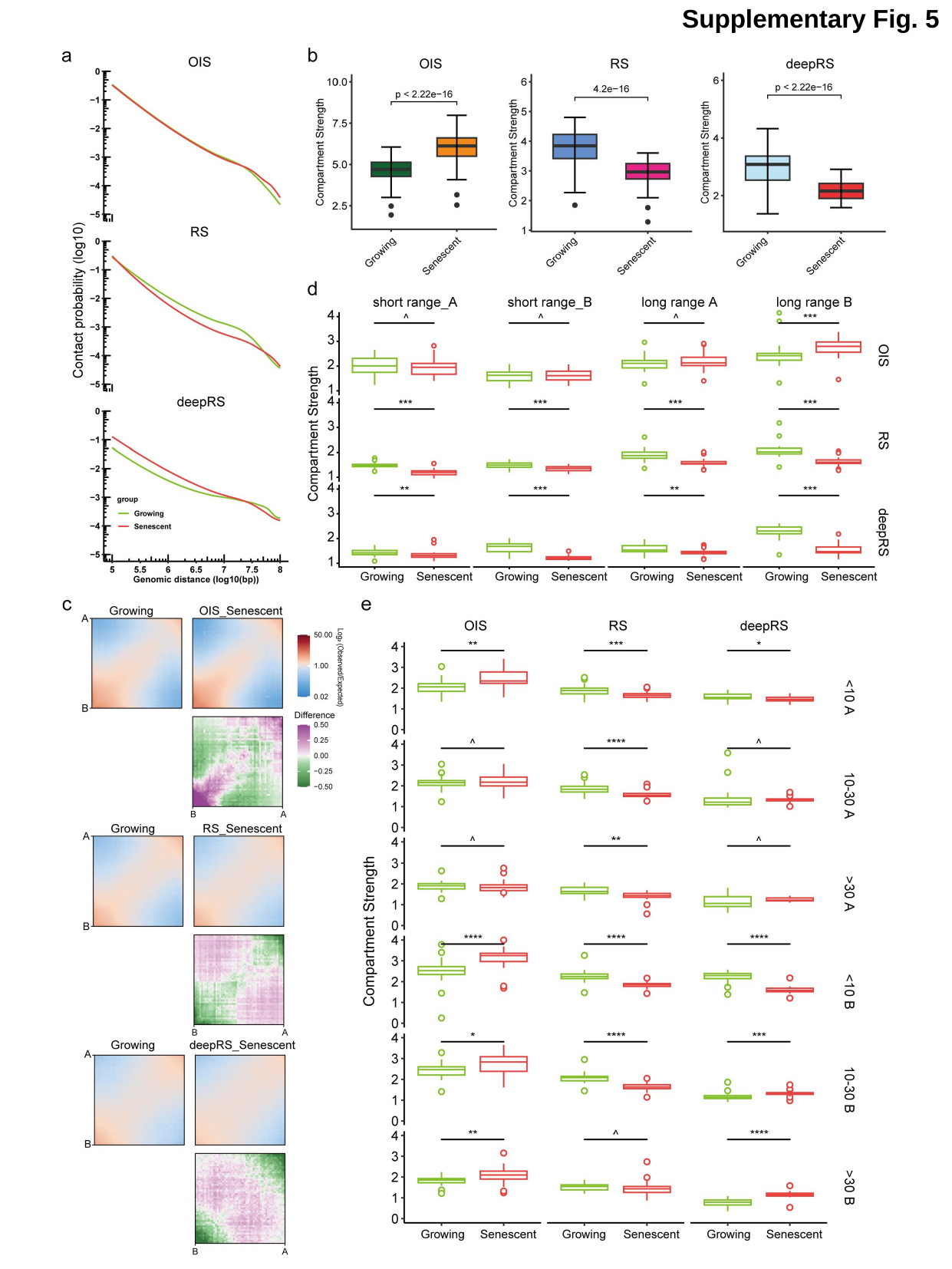

Supplementary Fig. 5

## Slide 9
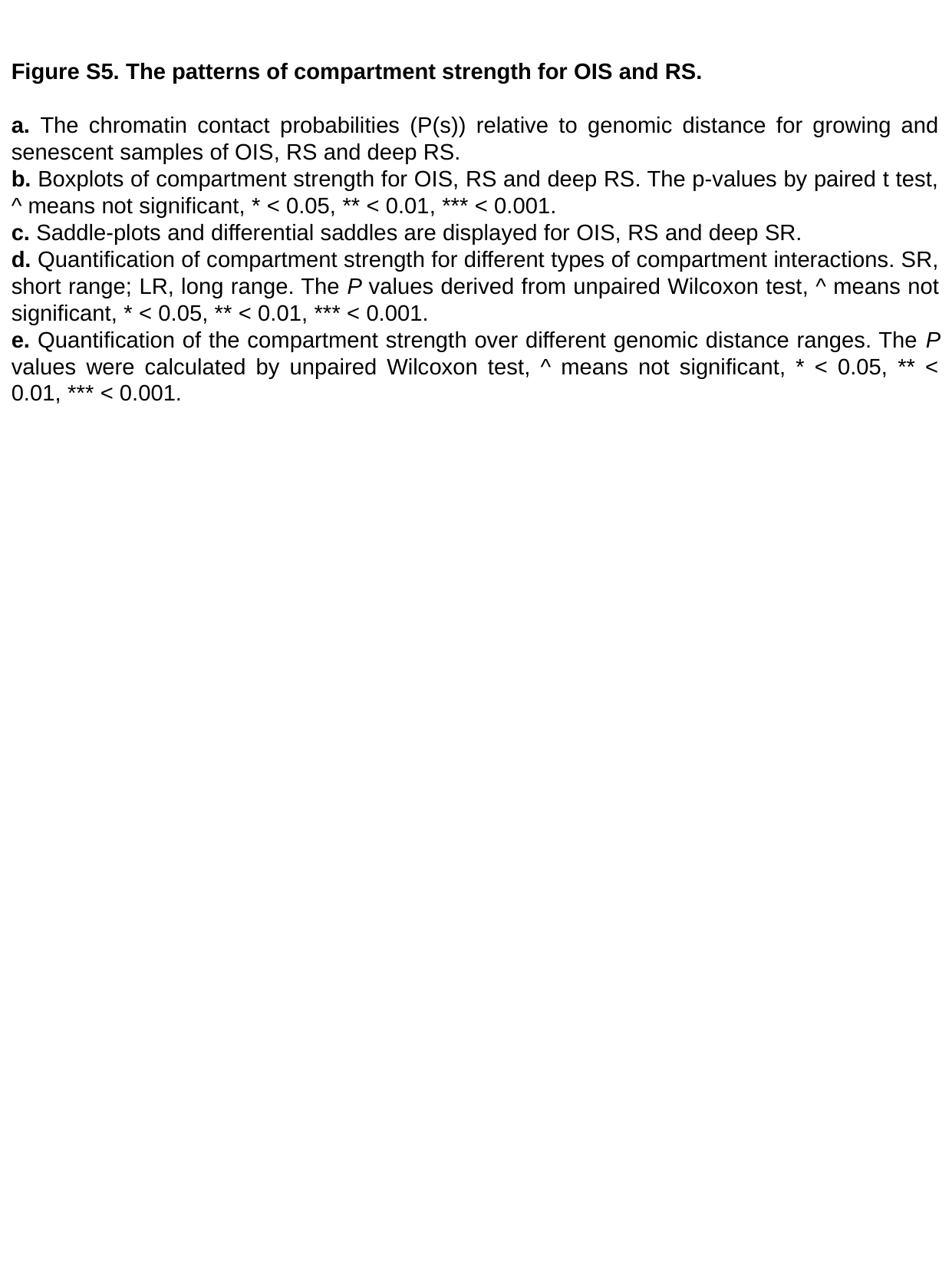

Figure S5. The patterns of compartment strength for OIS and RS.
a. The chromatin contact probabilities (P(s)) relative to genomic distance for growing and senescent samples of OIS, RS and deep RS.
b. Boxplots of compartment strength for OIS, RS and deep RS. The p-values by paired t test, ^ means not significant, * < 0.05, ** < 0.01, *** < 0.001.
c. Saddle-plots and differential saddles are displayed for OIS, RS and deep SR.
d. Quantification of compartment strength for different types of compartment interactions. SR, short range; LR, long range. The P values derived from unpaired Wilcoxon test, ^ means not significant, * < 0.05, ** < 0.01, *** < 0.001.
e. Quantification of the compartment strength over different genomic distance ranges. The P values were calculated by unpaired Wilcoxon test, ^ means not significant, * < 0.05, ** < 0.01, *** < 0.001.

## Slide 10
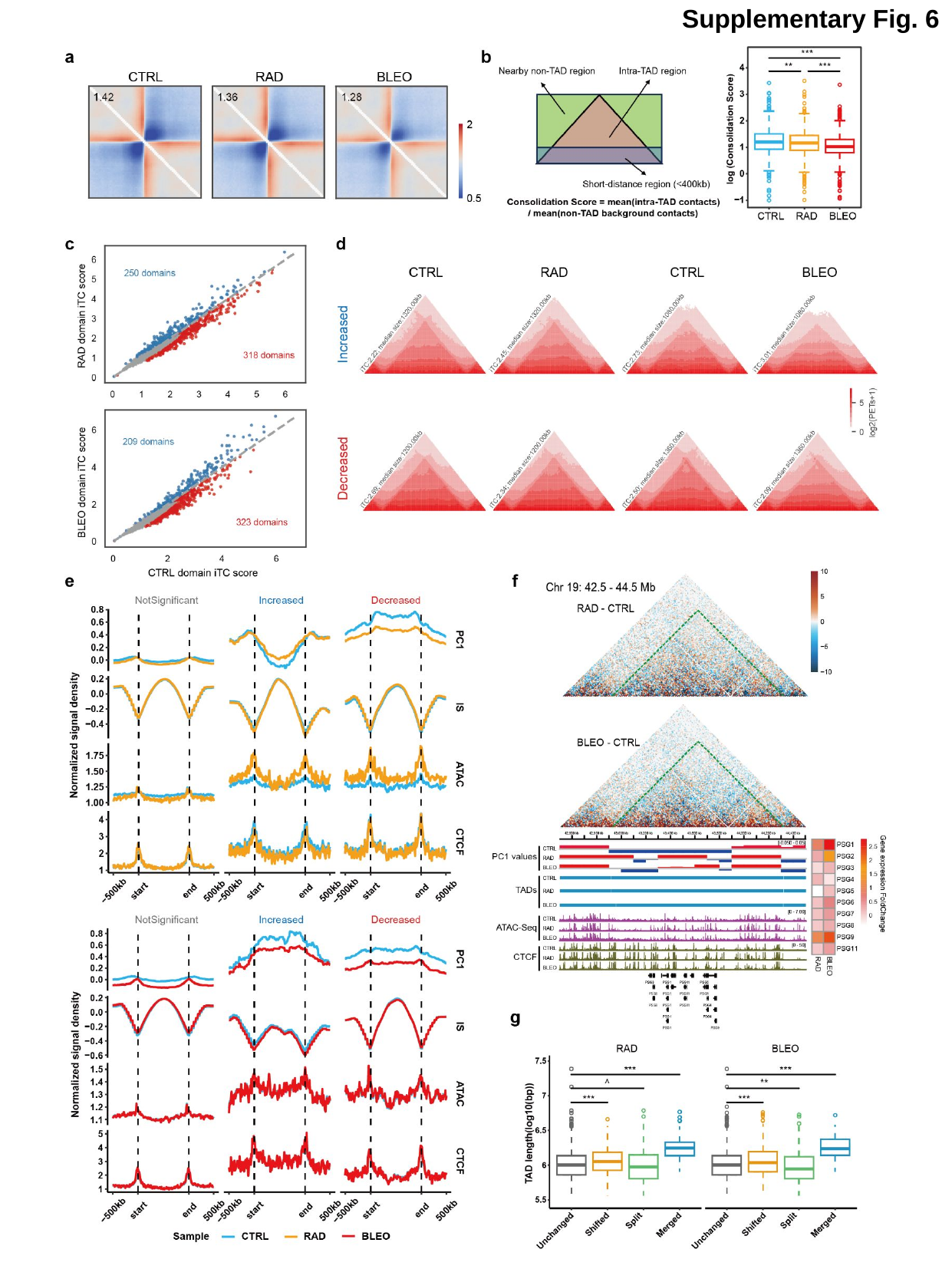

Supplementary Fig. 6

## Slide 11
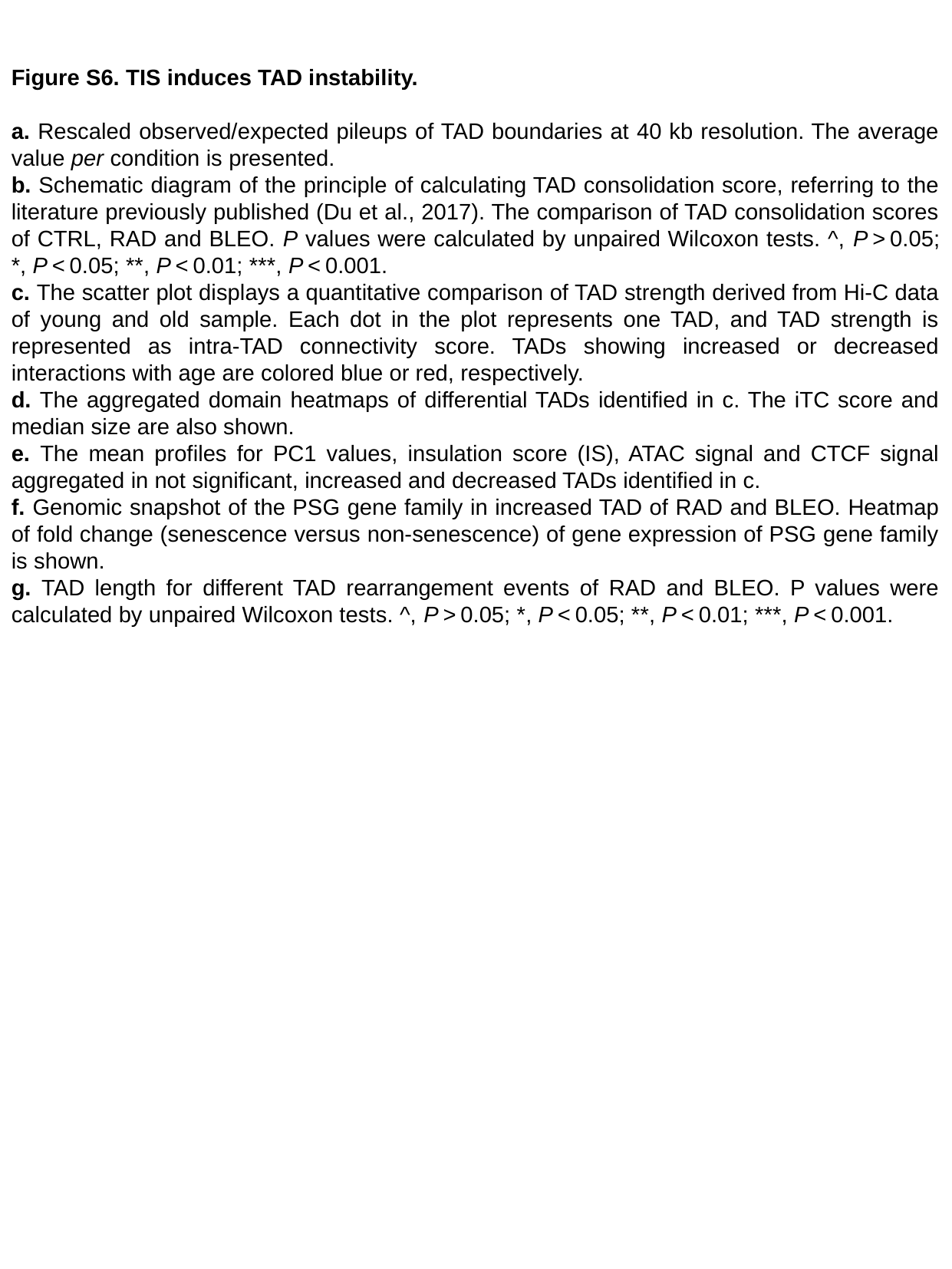

Figure S6. TIS induces TAD instability.
a. Rescaled observed/expected pileups of TAD boundaries at 40 kb resolution. The average value per condition is presented.
b. Schematic diagram of the principle of calculating TAD consolidation score, referring to the literature previously published (Du et al., 2017). The comparison of TAD consolidation scores of CTRL, RAD and BLEO. P values were calculated by unpaired Wilcoxon tests. ^, P > 0.05; *, P < 0.05; **, P < 0.01; ***, P < 0.001.
c. The scatter plot displays a quantitative comparison of TAD strength derived from Hi-C data of young and old sample. Each dot in the plot represents one TAD, and TAD strength is represented as intra-TAD connectivity score. TADs showing increased or decreased interactions with age are colored blue or red, respectively.
d. The aggregated domain heatmaps of differential TADs identified in c. The iTC score and median size are also shown.
e. The mean profiles for PC1 values, insulation score (IS), ATAC signal and CTCF signal aggregated in not significant, increased and decreased TADs identified in c.
f. Genomic snapshot of the PSG gene family in increased TAD of RAD and BLEO. Heatmap of fold change (senescence versus non-senescence) of gene expression of PSG gene family is shown.
g. TAD length for different TAD rearrangement events of RAD and BLEO. P values were calculated by unpaired Wilcoxon tests. ^, P > 0.05; *, P < 0.05; **, P < 0.01; ***, P < 0.001.

## Slide 12
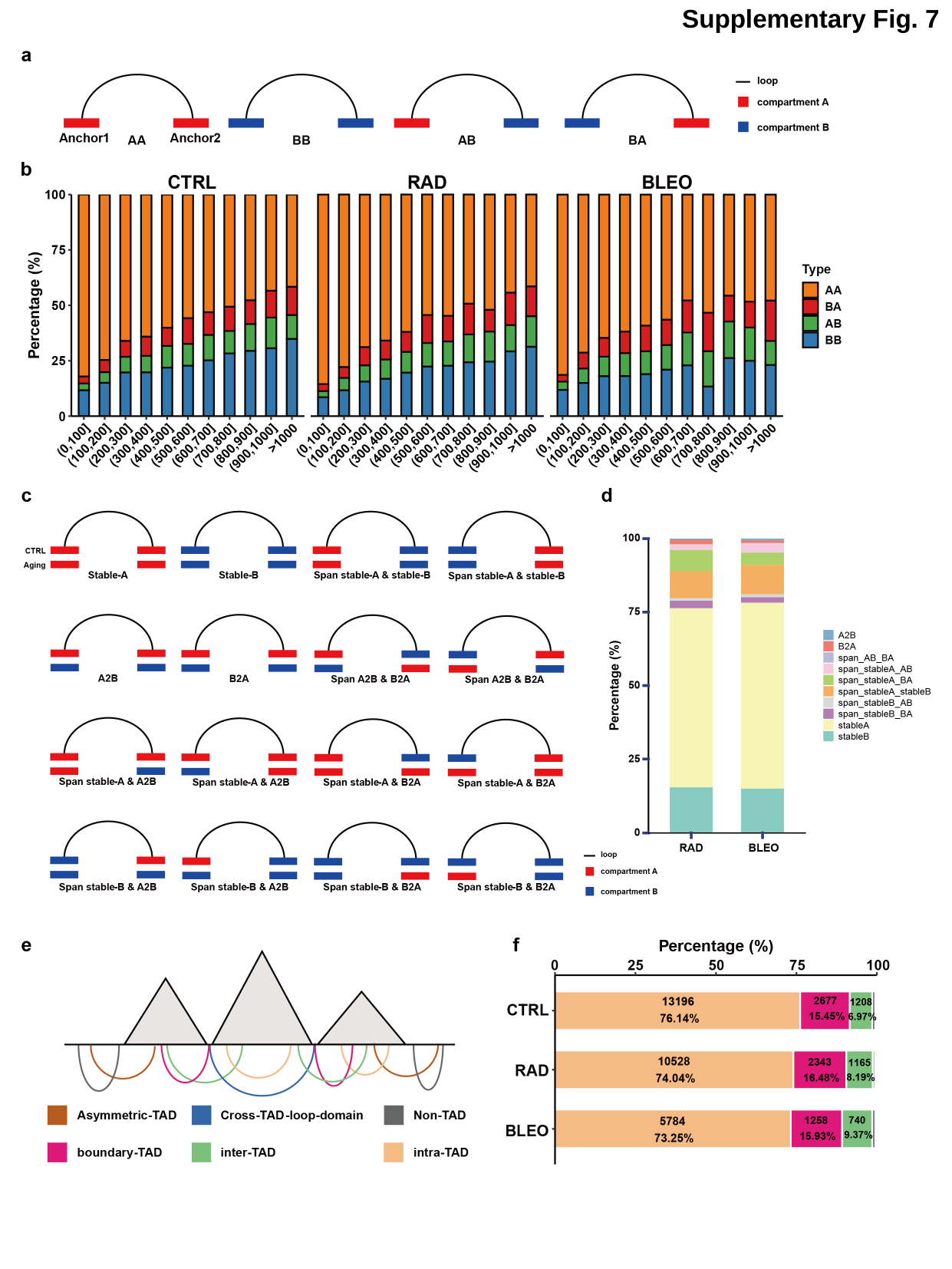

Supplementary Fig. 7

## Slide 13
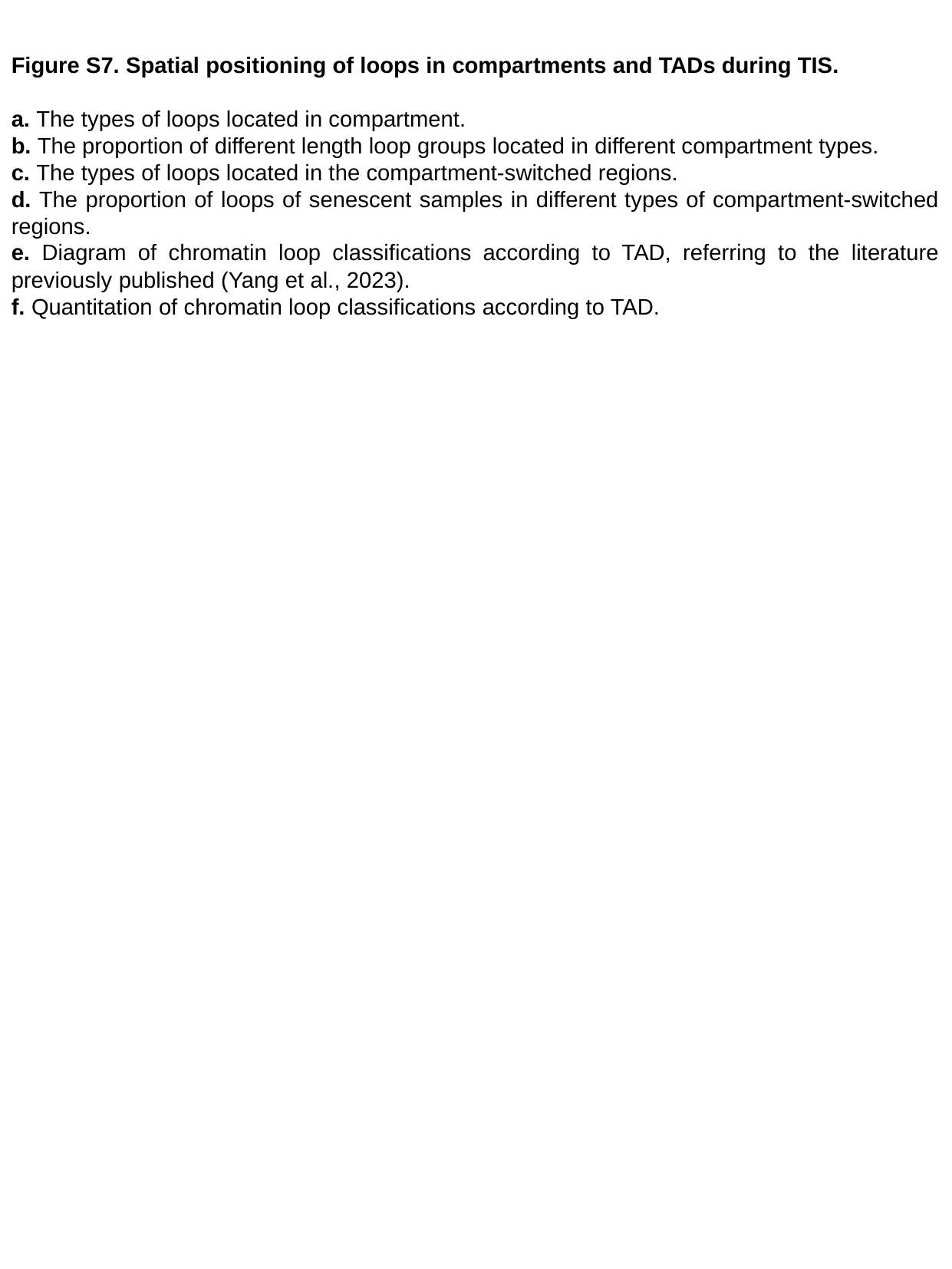

Figure S7. Spatial positioning of loops in compartments and TADs during TIS.
a. The types of loops located in compartment.
b. The proportion of different length loop groups located in different compartment types.
c. The types of loops located in the compartment-switched regions.
d. The proportion of loops of senescent samples in different types of compartment-switched regions.
e. Diagram of chromatin loop classifications according to TAD, referring to the literature previously published (Yang et al., 2023).
f. Quantitation of chromatin loop classifications according to TAD.

## Slide 14
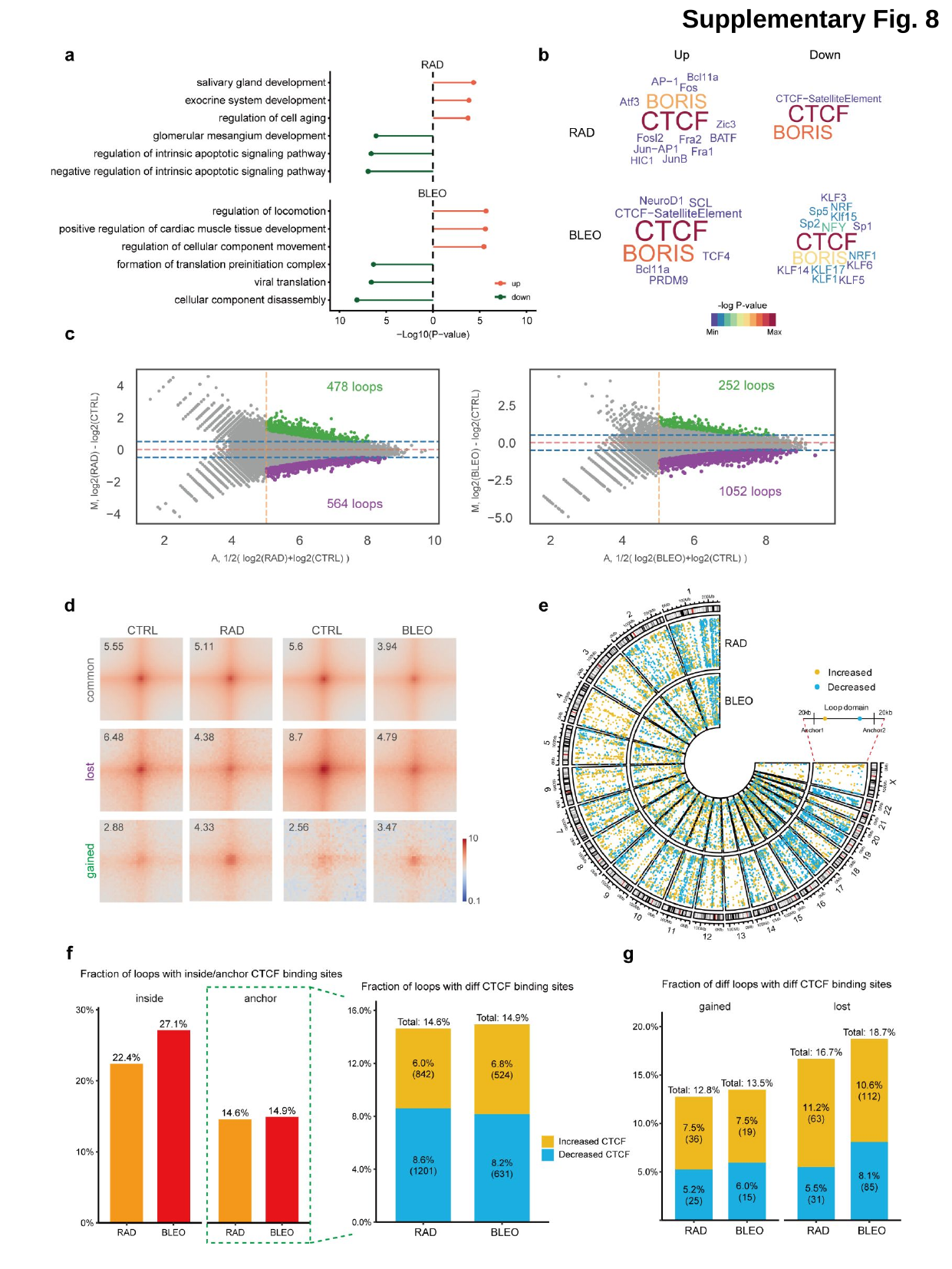

Supplementary Fig. 8

## Slide 15
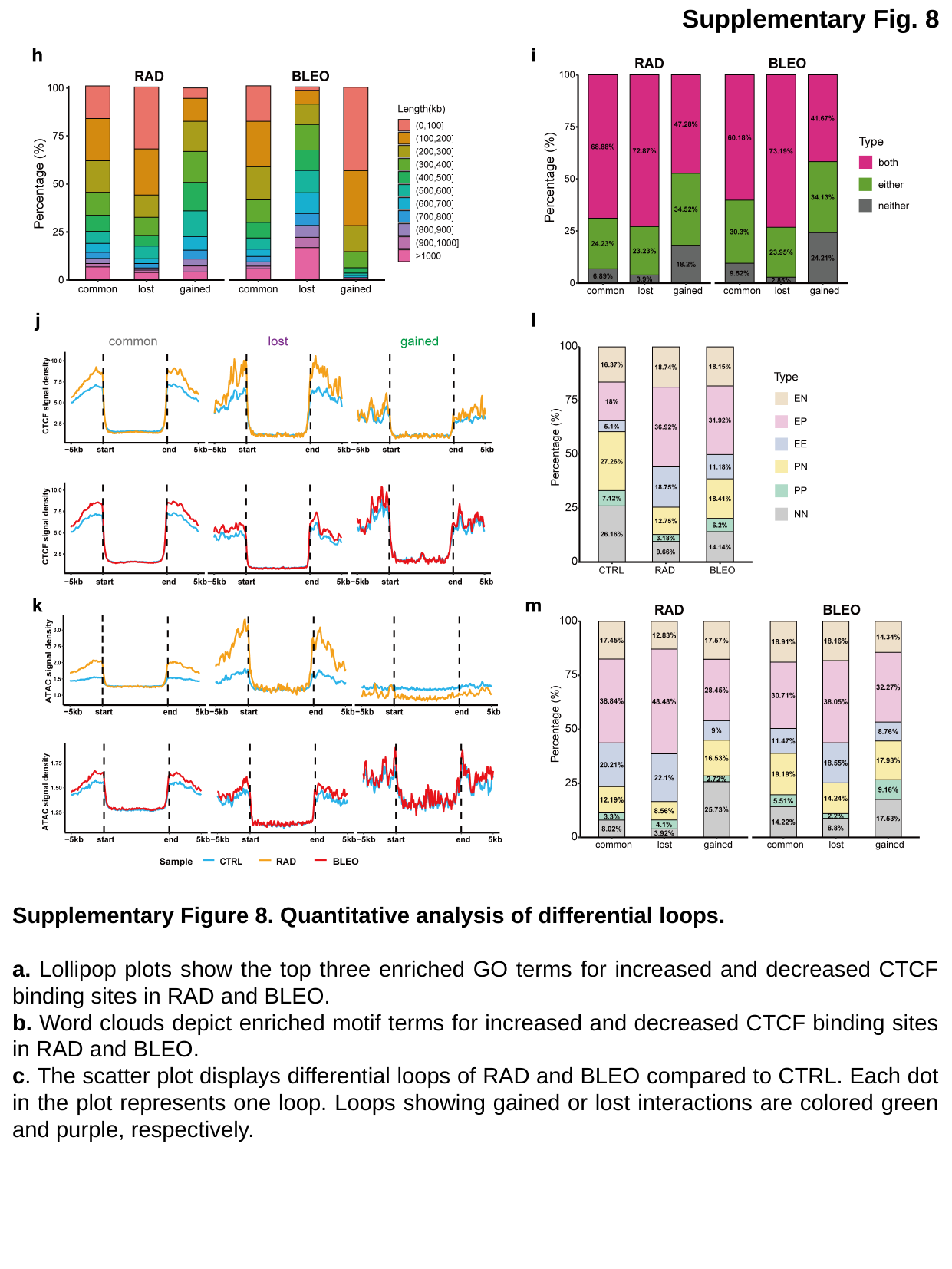

Supplementary Fig. 8
Supplementary Figure 8. Quantitative analysis of differential loops.
a. Lollipop plots show the top three enriched GO terms for increased and decreased CTCF binding sites in RAD and BLEO.
b. Word clouds depict enriched motif terms for increased and decreased CTCF binding sites in RAD and BLEO.
c. The scatter plot displays differential loops of RAD and BLEO compared to CTRL. Each dot in the plot represents one loop. Loops showing gained or lost interactions are colored green and purple, respectively.

## Slide 16
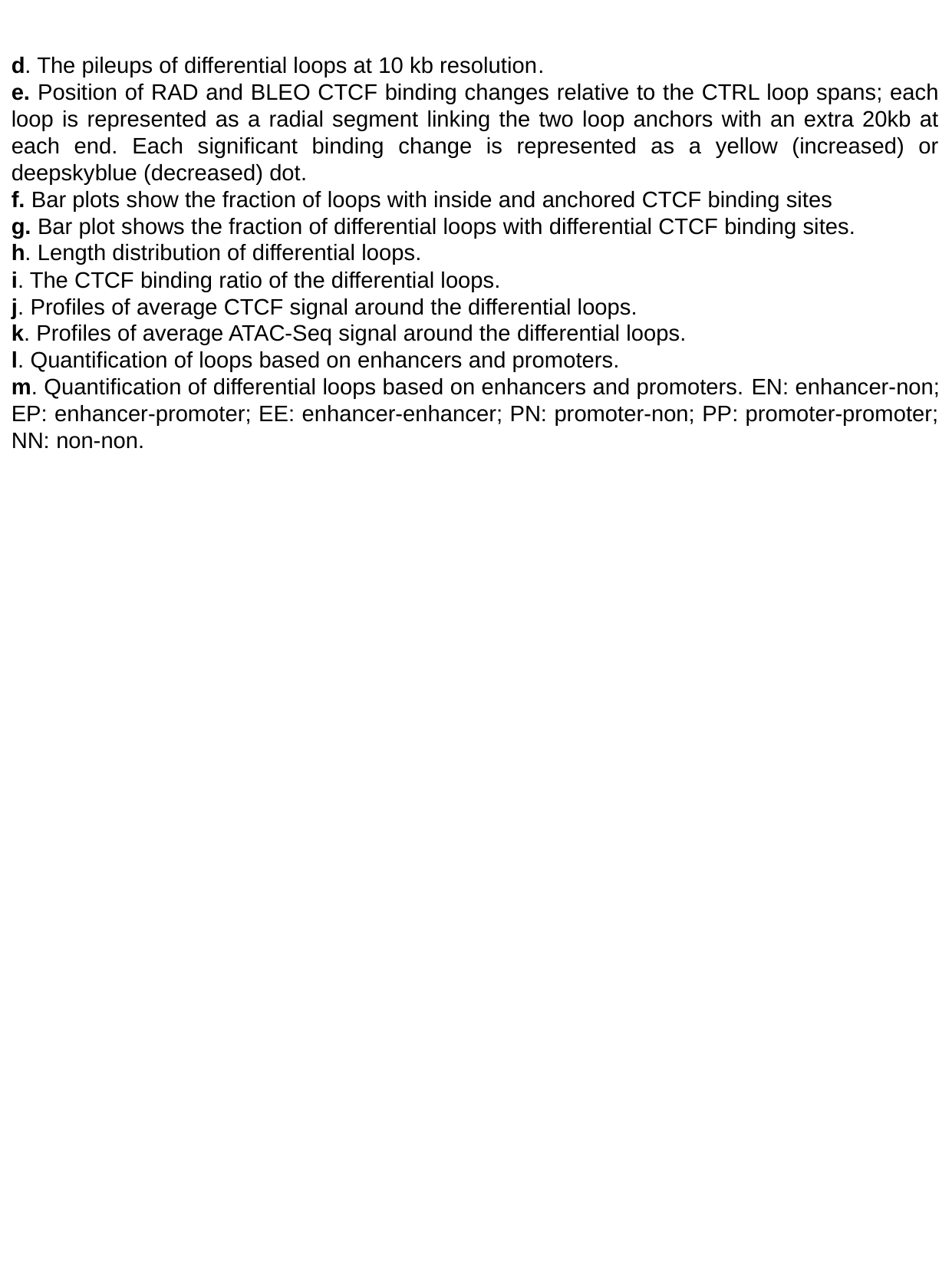

d. The pileups of differential loops at 10 kb resolution.
e. Position of RAD and BLEO CTCF binding changes relative to the CTRL loop spans; each loop is represented as a radial segment linking the two loop anchors with an extra 20kb at each end. Each significant binding change is represented as a yellow (increased) or deepskyblue (decreased) dot.
f. Bar plots show the fraction of loops with inside and anchored CTCF binding sites
g. Bar plot shows the fraction of differential loops with differential CTCF binding sites.
h. Length distribution of differential loops.
i. The CTCF binding ratio of the differential loops.
j. Profiles of average CTCF signal around the differential loops.
k. Profiles of average ATAC-Seq signal around the differential loops.
l. Quantification of loops based on enhancers and promoters.
m. Quantification of differential loops based on enhancers and promoters. EN: enhancer-non; EP: enhancer-promoter; EE: enhancer-enhancer; PN: promoter-non; PP: promoter-promoter; NN: non-non.

## Slide 17
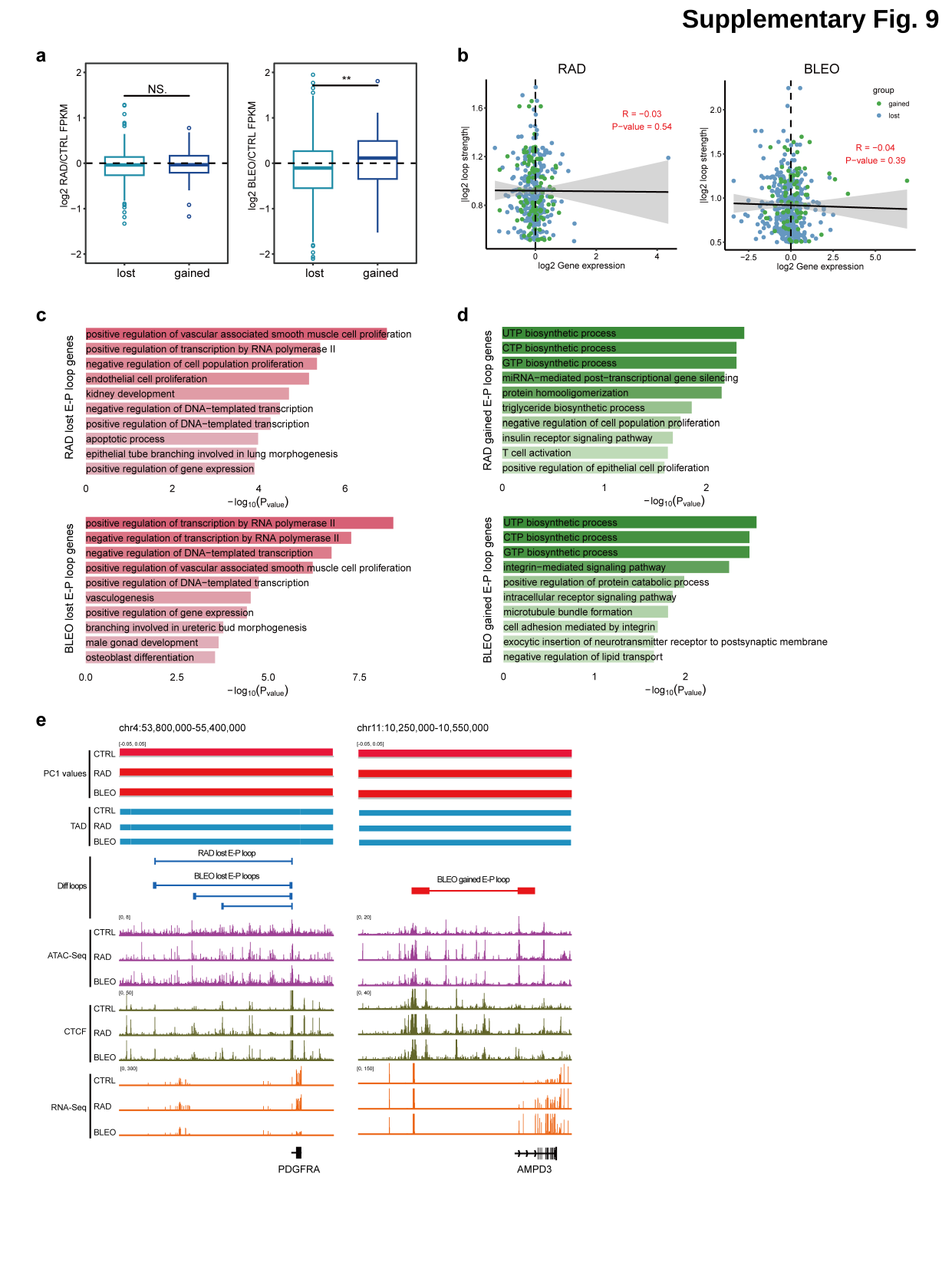

Supplementary Fig. 9

## Slide 18
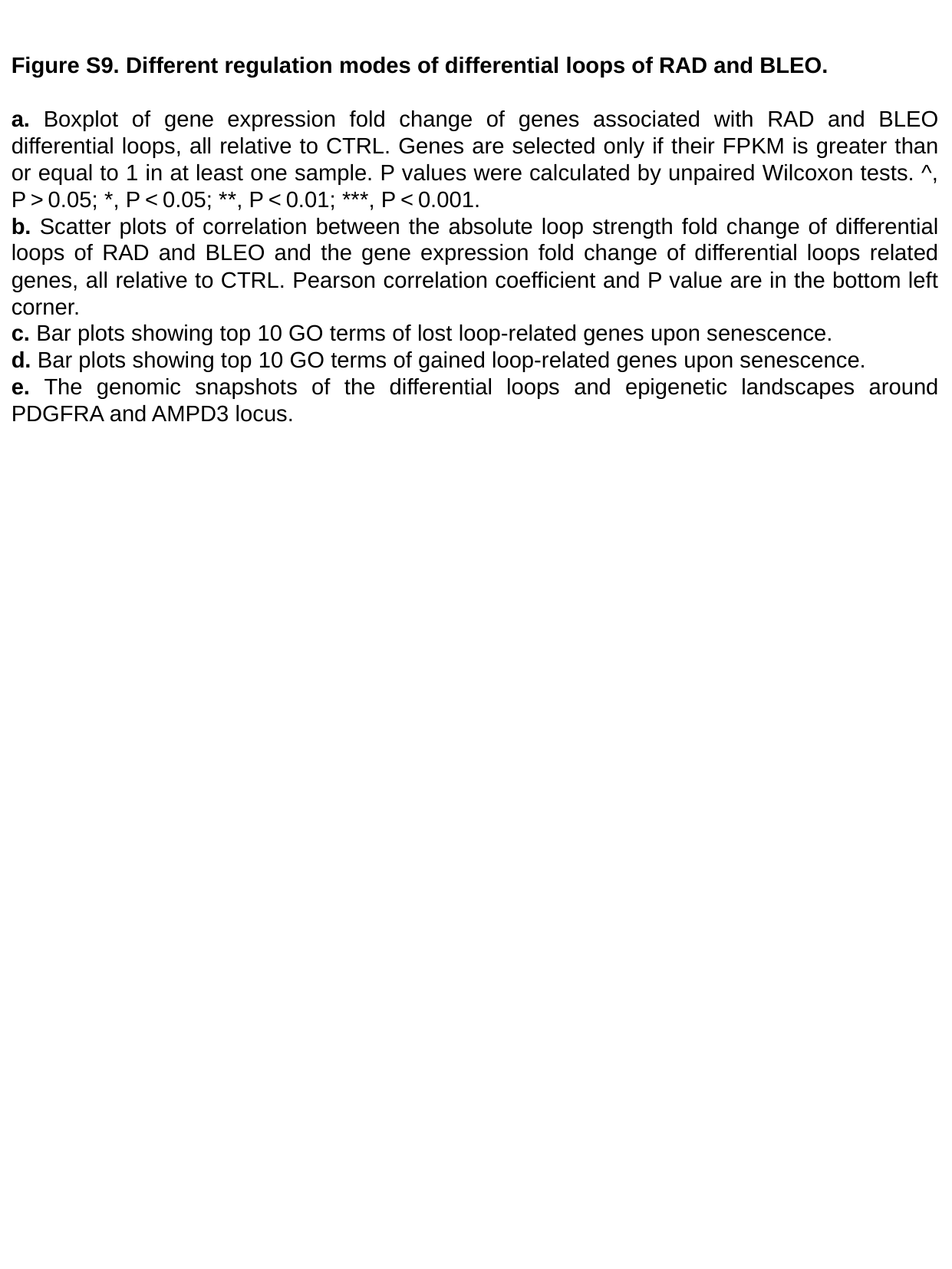

Figure S9. Different regulation modes of differential loops of RAD and BLEO.
a. Boxplot of gene expression fold change of genes associated with RAD and BLEO differential loops, all relative to CTRL. Genes are selected only if their FPKM is greater than or equal to 1 in at least one sample. P values were calculated by unpaired Wilcoxon tests. ^, P > 0.05; *, P < 0.05; **, P < 0.01; ***, P < 0.001.
b. Scatter plots of correlation between the absolute loop strength fold change of differential loops of RAD and BLEO and the gene expression fold change of differential loops related genes, all relative to CTRL. Pearson correlation coefficient and P value are in the bottom left corner.
c. Bar plots showing top 10 GO terms of lost loop-related genes upon senescence.
d. Bar plots showing top 10 GO terms of gained loop-related genes upon senescence.
e. The genomic snapshots of the differential loops and epigenetic landscapes around PDGFRA and AMPD3 locus.

## Slide 19
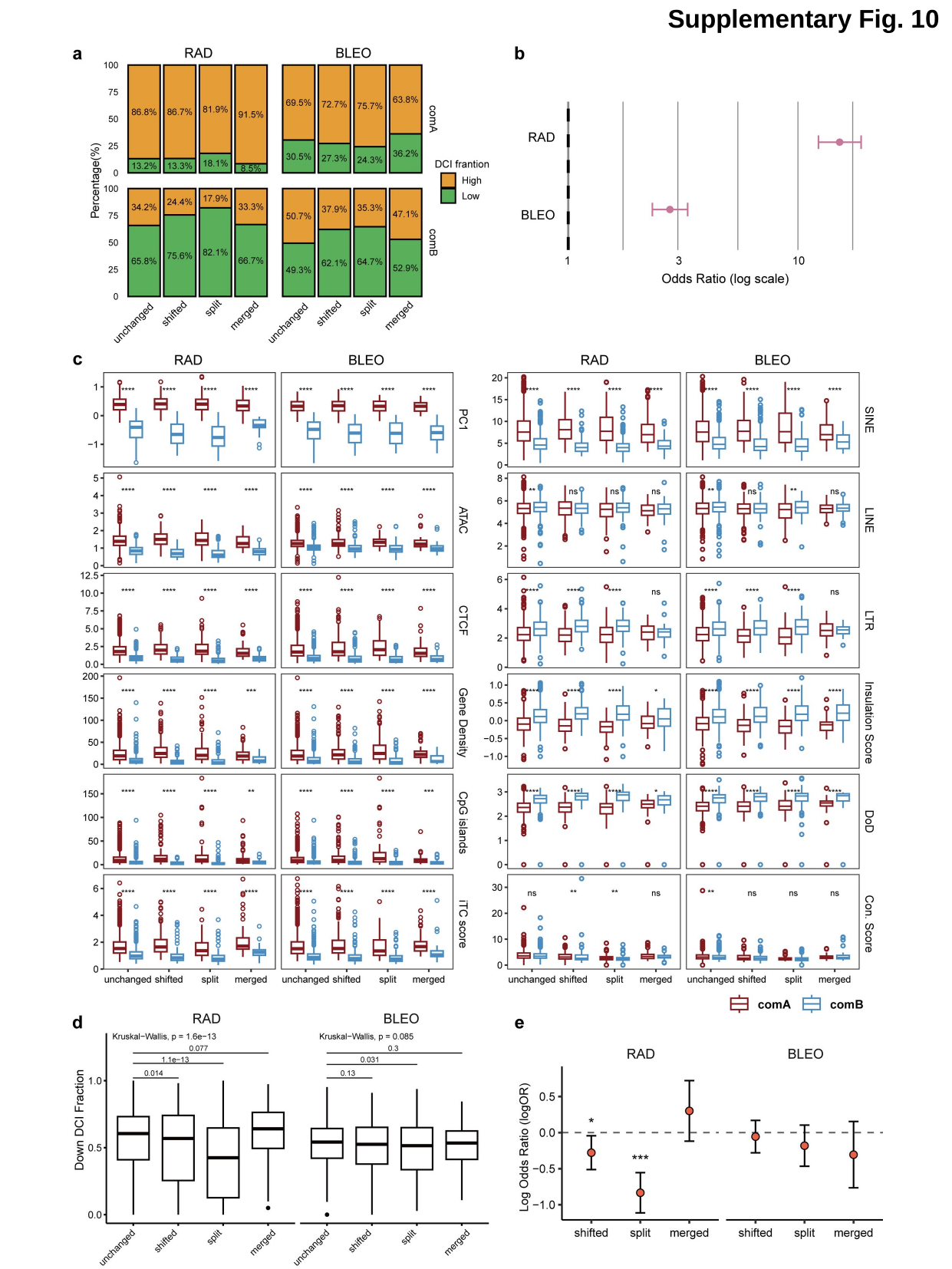

Supplementary Fig. 10

## Slide 20
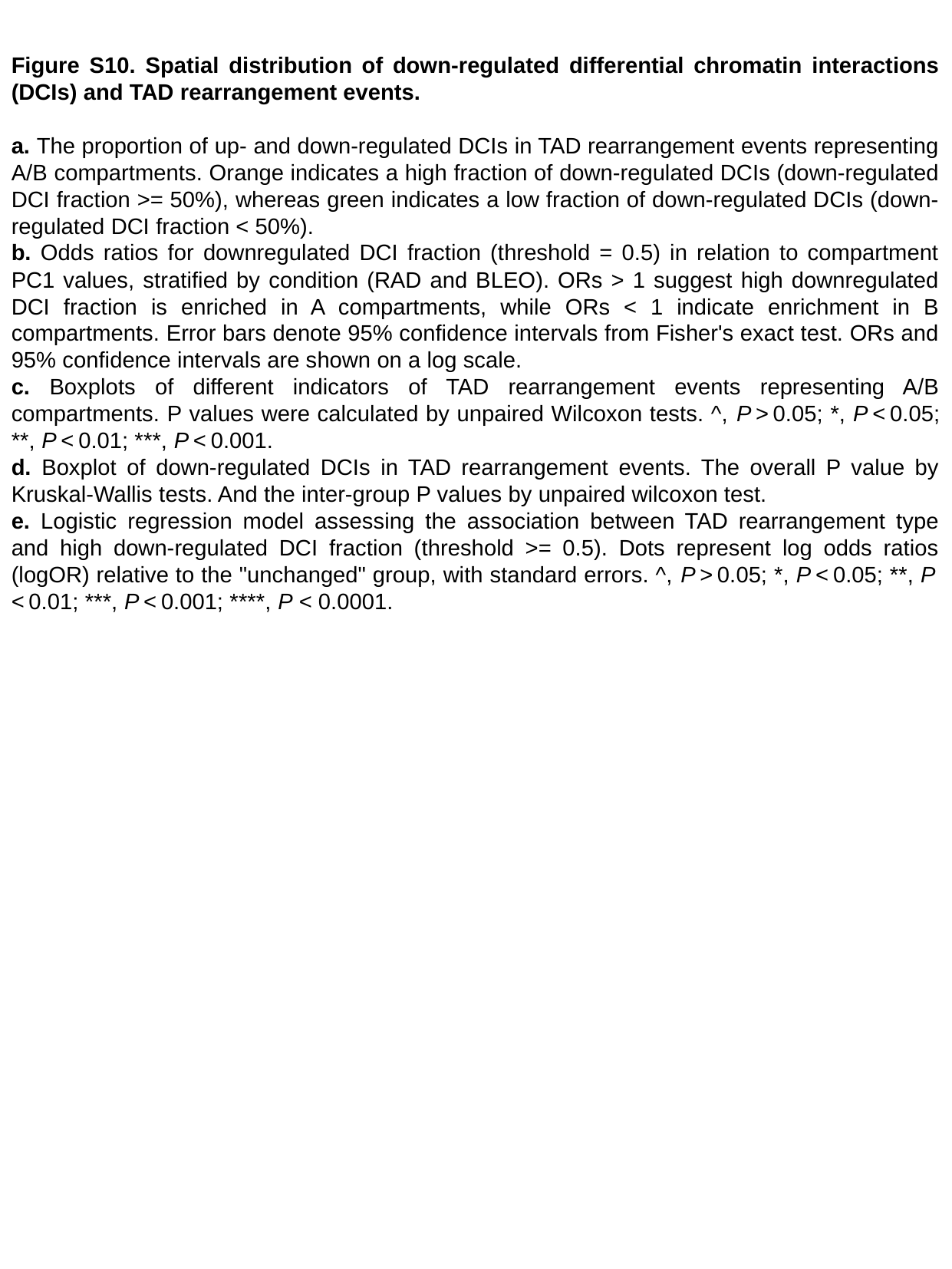

Figure S10. Spatial distribution of down-regulated differential chromatin interactions (DCIs) and TAD rearrangement events.
a. The proportion of up- and down-regulated DCIs in TAD rearrangement events representing A/B compartments. Orange indicates a high fraction of down-regulated DCIs (down-regulated DCI fraction >= 50%), whereas green indicates a low fraction of down-regulated DCIs (down-regulated DCI fraction < 50%).
b. Odds ratios for downregulated DCI fraction (threshold = 0.5) in relation to compartment PC1 values, stratified by condition (RAD and BLEO). ORs > 1 suggest high downregulated DCI fraction is enriched in A compartments, while ORs < 1 indicate enrichment in B compartments. Error bars denote 95% confidence intervals from Fisher's exact test. ORs and 95% confidence intervals are shown on a log scale.
c. Boxplots of different indicators of TAD rearrangement events representing A/B compartments. P values were calculated by unpaired Wilcoxon tests. ^, P > 0.05; *, P < 0.05; **, P < 0.01; ***, P < 0.001.
d. Boxplot of down-regulated DCIs in TAD rearrangement events. The overall P value by Kruskal-Wallis tests. And the inter-group P values by unpaired wilcoxon test.
e. Logistic regression model assessing the association between TAD rearrangement type and high down-regulated DCI fraction (threshold >= 0.5). Dots represent log odds ratios (logOR) relative to the "unchanged" group, with standard errors. ^, P > 0.05; *, P < 0.05; **, P < 0.01; ***, P < 0.001; ****, P < 0.0001.

## Slide 21
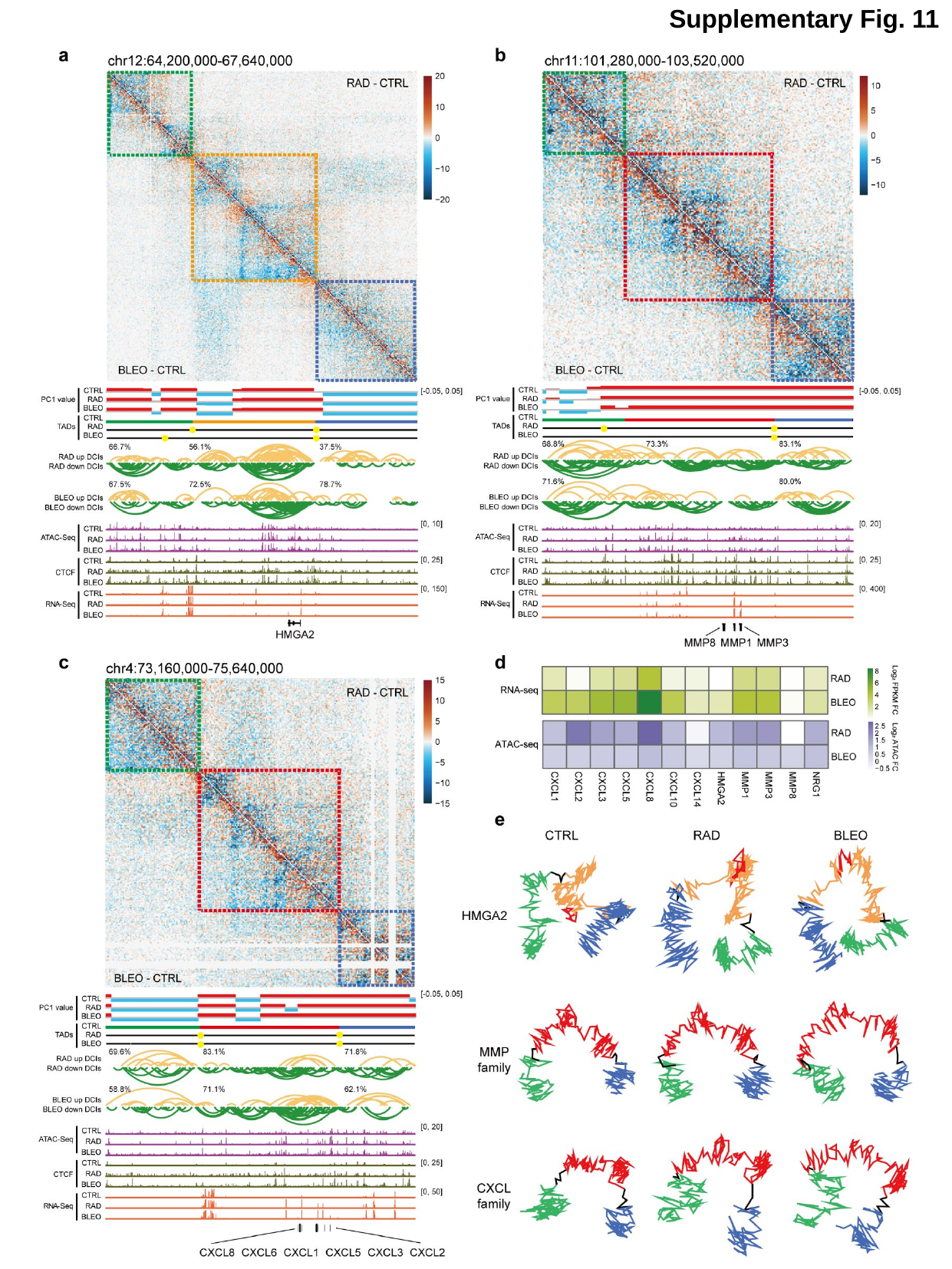

Supplementary Fig. 11

## Slide 22
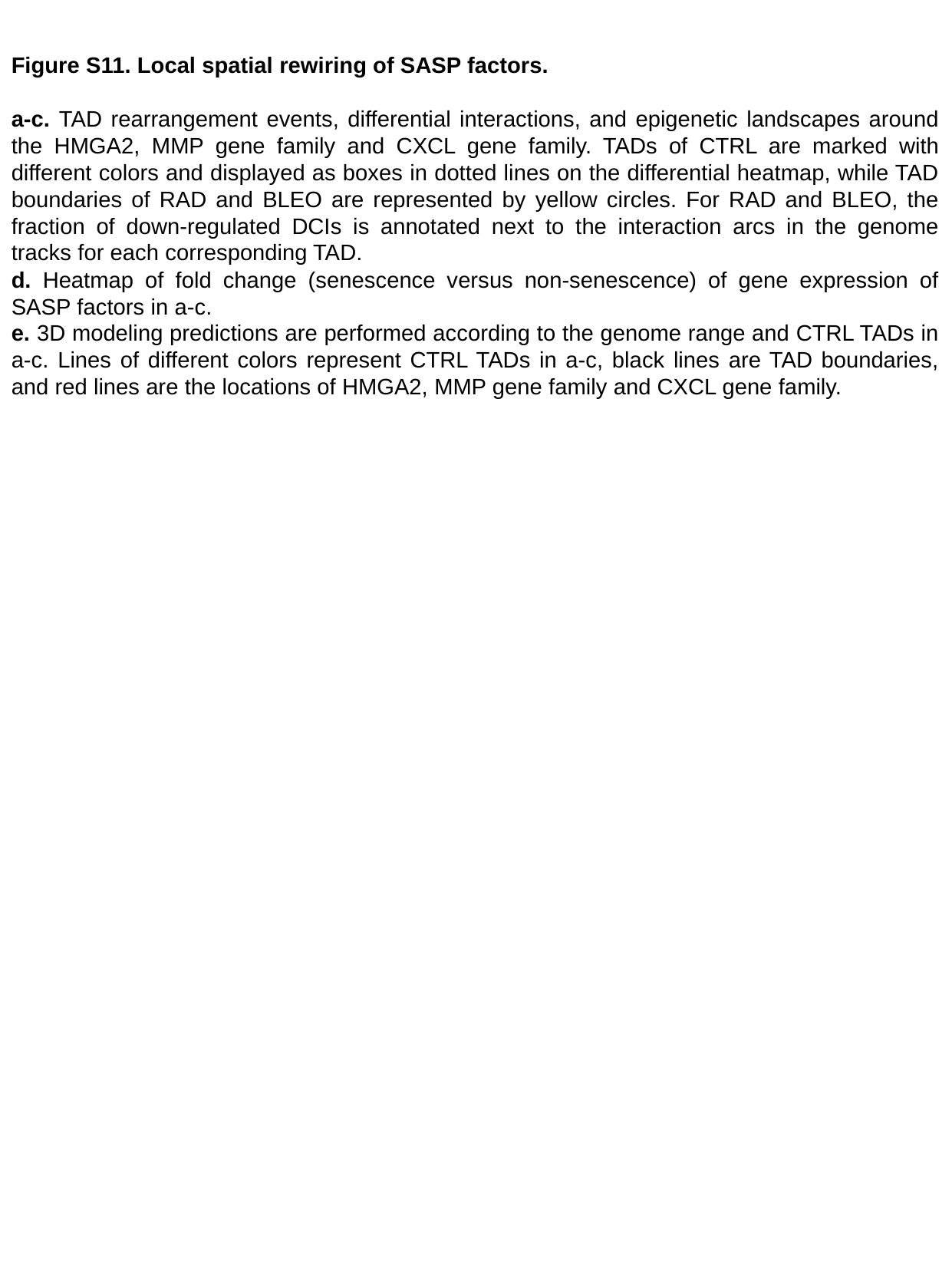

Figure S11. Local spatial rewiring of SASP factors.
a-c. TAD rearrangement events, differential interactions, and epigenetic landscapes around the HMGA2, MMP gene family and CXCL gene family. TADs of CTRL are marked with different colors and displayed as boxes in dotted lines on the differential heatmap, while TAD boundaries of RAD and BLEO are represented by yellow circles. For RAD and BLEO, the fraction of down-regulated DCIs is annotated next to the interaction arcs in the genome tracks for each corresponding TAD.
d. Heatmap of fold change (senescence versus non-senescence) of gene expression of SASP factors in a-c.
e. 3D modeling predictions are performed according to the genome range and CTRL TADs in a-c. Lines of different colors represent CTRL TADs in a-c, black lines are TAD boundaries, and red lines are the locations of HMGA2, MMP gene family and CXCL gene family.

## Slide 23
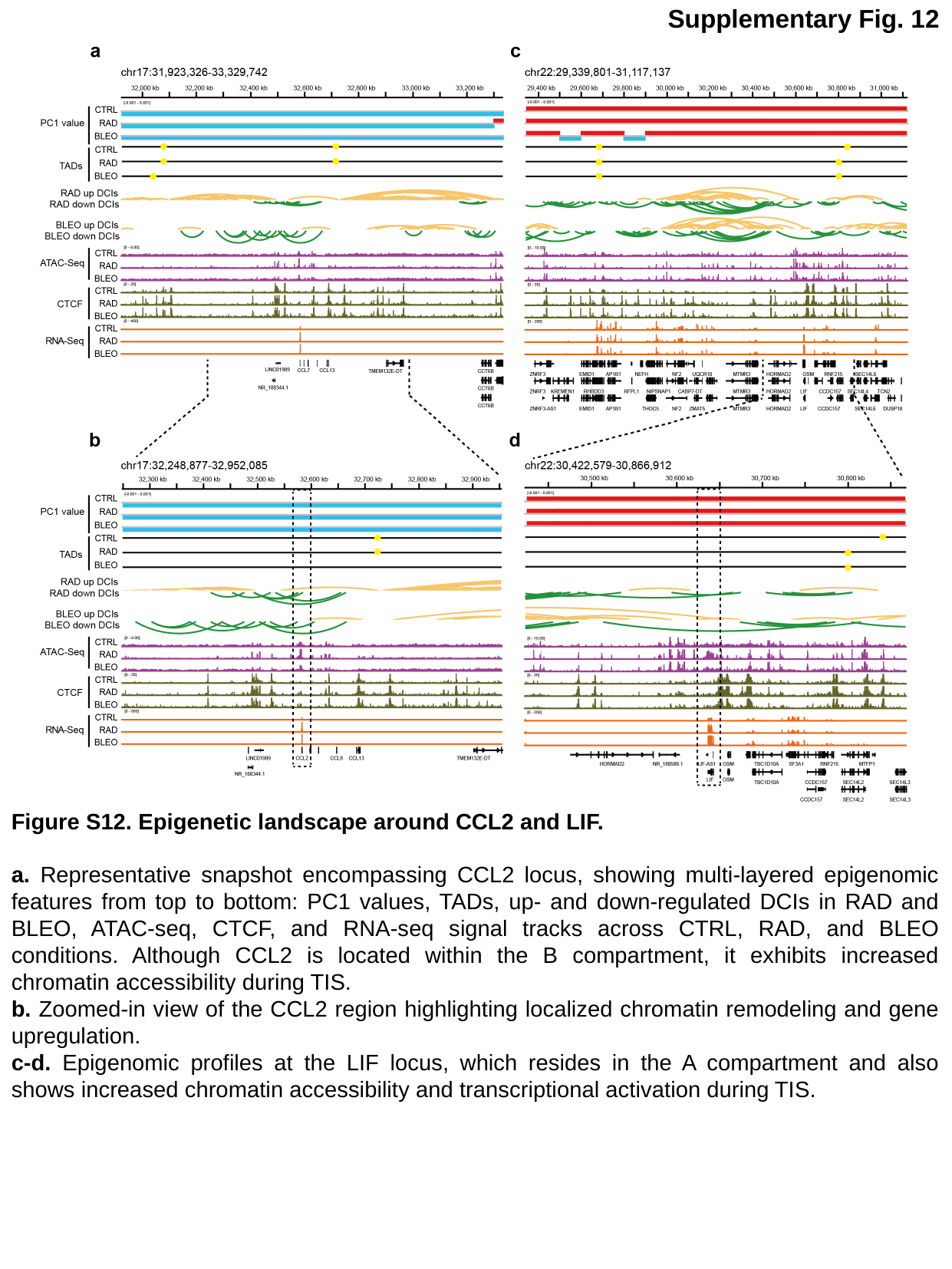

Supplementary Fig. 12
Figure S12. Epigenetic landscape around CCL2 and LIF.
a. Representative snapshot encompassing CCL2 locus, showing multi-layered epigenomic features from top to bottom: PC1 values, TADs, up- and down-regulated DCIs in RAD and BLEO, ATAC-seq, CTCF, and RNA-seq signal tracks across CTRL, RAD, and BLEO conditions. Although CCL2 is located within the B compartment, it exhibits increased chromatin accessibility during TIS.
b. Zoomed-in view of the CCL2 region highlighting localized chromatin remodeling and gene upregulation.
c-d. Epigenomic profiles at the LIF locus, which resides in the A compartment and also shows increased chromatin accessibility and transcriptional activation during TIS.
